# Recreational beneficiaries and their landscape dependencies across national estuary program sites: Tillamook Bay (OR) and Tampa Bay (FL), USA

**DOI:** 10.1080/26395916.2023.2276756

**Published:** 2023-11-15

**Authors:** Chanda Jones Littles, Nathaniel S. Lewis, Theodore H. DeWitt, Matthew C. Harwell

**Affiliations:** a Oak Ridge Institute for Science and Education (ORISE) Fellow, Environmental Protection Agency - Pacific Ecological Systems Division, Center for Public Health and Environmental Assessment, Newport, OR, USA; b Environmental Resources Branch, U.S. Army Corps of Engineers, Portland, OR, USA; c Coastal Oregon Marine Experiment Station (COMES), Oregon State University, Newport, OR, USA; d Environmental Protection Agency - Pacific Ecological Systems Division, Center for Public Health and Environmental Assessment, Newport, OR, USA

**Keywords:** Coastal recreation, ecosystem service provision, cultural ecosystem services, recreational services, ecosystem-based management

## Abstract

This study aims to characterize the value associated with nature-based recreational opportunities and identify estuarine attributes most valued by users. With the National Ecosystem Service Classification System as a framework, we assessed the relationship between recreational beneficiary subclasses and ecological end-products available to beneficiaries in Tillamook Bay, OR, and Tampa Bay, FL estuaries. We used the InVEST recreation model to assess the spatial distribution and intensity of recreation in both estuaries, then inform site selection in subsequent analyses. We evaluated photo content and collected observational data at sites with the highest utilization. Surveys of location attributes helped determine the availability of ecosystem service ecological end-products. Ordination techniques were employed to evaluate similarities in natural and human-made attributes across stations and establish groups of stations that could offer comparable recreational experiences. Recreational ‘experiencers and viewers’ were the dominant beneficiary group, as they took the most photos and were most often encountered during passive onsite observations. Composite features (e.g. viewscapes) were the predominant ecological end-products. Counter to hypothesized outcomes, there was no detectable difference in the number of recreational beneficiaries predicted between estuaries after accounting for site-scale variability. Locations with multiple natural and human-made attributes, including access points, had more recreational users. Onsite observations also revealed a potential need for more safe and equitable access options in high-use locations. Findings highlight the importance of recreational ‘experiencers and viewers’ valuing habitat mosaics, even across vastly different geographical settings. This exploration of how humans derive well-being from coastal landscapes is crucial to ecosystem-based management.

## 1. Introduction

Estuaries provide many direct and indirect benefits to people while also serving as hotspots for biodiversity as they coalesce properties of terrestrial, freshwater and marine environments. As with many other coastal environments, humans are part of the larger ecosystem, and our decisions affect the quality and quantity of estuarine resources. Integrating apparent human uses into ecosystem-based management and identifying ways to improve responsible, sustained use can lead to better outcomes for both the environment and people (Granek et al. 2010; Jordan et al. 2010; Yoskowitz and Russell 2015; Boerema and Meire 2017; O’Higgins et al. 2020). Learning how communities value and interact with estuarine landscapes is the first step in working toward this end goal. A focus on final ecosystem goods and services (FEGS), or those aspects of nature that are directly utilized by people (Boyd and Banzhaf 2007; Landers and Nahlik 2013; DeWitt et al. 2020), can aid in determining natural estuarine attributes that contribute to human well-being and evaluating how changes in those attributes may ultimately affect FEGS availability (Daw et al. 2016; Pouso et al. 2018). An explicit consideration of how FEGS flow from the natural environment to human production processes or aspects of well-being can aid in more holistic management approaches (EPA, United States Environmental Protection Agency 2015; Yee et al. 2017).

Coastal estuaries host a variety of sub-ecosystems including wetlands, beaches, transitional waters, intertidal areas and various other habitats that contribute to an array of provisioning, regulating and cultural ecosystem services (Barbier et al. 2011). Several studies have assessed the link between coastal habitats and food provision, water purification, property protection, recreation and tourism (Liquete et al. 2013). However, the relationship between ecosystem services and human well-being is often non-linear and complex such that habitat degradation does not always result in observable declines to indicators of well-being (Daw et al. 2016; De La Cruz 2021). Typical indicators for recreational ecosystem services include income from tourism, the number of visitors to a location, travel costs and willingness to pay (Liquete et al. 2013). These can be helpful in regional planning but may not provide sufficient information about underlying habitat conditions that sustain human-use or are most valued. Recreational user groups commonly assessed in FEGS studies (e.g. boaters, swimmers, fishermen, etc.) may not adequately account for more passive or indirect users or uses. This study delves into these questions by evaluating the aspects of the environment most featured in perception-based data and by observing how recreational users interact with specific locations across two estuaries.

In the context of recreational FEGS, coastal resource managers can design targeted outreach based on locations facilitating greater FEGS use, land stewards may use FEGS to determine what opportunities exist for improving site conditions at high-use areas, and resource agencies may prioritize FEGS-rich locations for encouraging citizen science and community involvement in data collection and monitoring, especially when local residents are the dominant users. Within the United States, the U.S. Environmental Protection Agency (EPA) established National Estuary Programs (NEPs) to help monitor estuarine resources and work directly with stakeholders to effectively manage these critical ecosystems (Imperial et al. 1993). The 28 NEP sites provide an ideal opportunity for taking a closer look at natural landscape attributes most closely aligned with FEGS (e.g. Yee et al. 2019). In fact, some NEP programs have used information about ecosystem services values and valuation to set restoration goals and communicate effectively with stakeholders (Martin 2014).

This manuscript builds on the growing evidence linking FEGS to various coastal and estuarine habitats (Littles et al. 2018), with a focus on shared attributes that may underlie a common suite of coastal recreational beneficiary subclasses, regardless of the estuary. Using EPA’s National Ecosystem Services Classification System (NESCS; Newcomer-Johnson et al. 2020) as a framework, we give explicit consideration to how FEGS flow from the natural environment (i.e. where they are produced) to where they are used by people to improve their well-being (EPA, United States Environmental Protection Agency 2015). The NESCS structure essentially breaks down the flow of FEGS into supply and demand. The supply culminates in a suite of ecological end products (EEP) (i.e. biophysical attributes) from the environment that provide benefits to people, and the demand (or use of EEPs) is ultimately determined by those people (Newcomer-Johnson et al. 2020). FEGS is the combination of an EEP and end-user (Table 1). This classification scheme recognizes EEPs as necessary inputs in getting to a final ecosystem service, which also encompasses a consideration of how those EEPs are being used and by whom. We focused on recreational users of estuaries and utilized multiple tools to analyze spatio-temporal patterns in recreational use. We had several hypotheses for how data sources (i.e. photos versus onsite observations), estuary setting and location attributes would shape FEGS:

**H**_**0**_**1:** *Flickr images will show a greater diversity of recreational users in Tampa Bay than Tillamook Bay*. [Reasoning – Tampa Bay is a much larger system offering a potentially wider array of recreational opportunities].

**H**_**0**_**2:** *Dominant recreational beneficiaries will differ between Tampa and Tillamook bays*. [Reasoning – The physical characteristics and geographical setting of each bay likely cater to a slightly different suite of recreational activities].

**H**_**0**_**3:** *Tampa Bay will have more recreational users observed than Tillamook Bay, but the diversity of recreational beneficiaries will be comparable*. [Reasoning – Tampa Bay is in a more urban watershed supporting a greater number of local residents and tourists available to engage in recreational activities].

**H**_**0**_**4:** *Location (sites and stations) will account for significant variability in the number and suite of recreational beneficiaries*. [Reasoning – The site-scale features will dictate the user experience and EEPs available, more so than larger landscape-scale characteristics].

**H**_**0**_**5:** *Stations with more attributes will correspond with greater numbers of recreational uses and beneficiaries*. [Reasoning – Greater site attributes equate to more or different opportunities for users to interface with the environment].

**H**_**0**_**6:** *Stations with similar attributes will have a similar portfolio of recreational users*. [Reasoning – Site-scale attributes largely dictate the recreationalexperience and sites with common features support similar uses].

**H**_**0**_**7:** *Onsite user observations will capture a greater diversity of recreational beneficiary subclasses than Flickr data*. [Reasoning – Flickr images are more likely to reflect recreational activities that are amenable to a user pausing and taking a photo. Many individuals may choose to refrain from taking photos while on the water or while actively recreating (e.g. kayaking, surfing, swimming, etc.)].

This study combines information from multiple data sources for a comprehensive look at both direct and indirect recreational use, the variation across coastal locations, and how relationships may change given the regional setting. By comparing recreational FEGS and landscape features across estuaries, we capitalized on existing relationships between stakeholders, scientists and decision-makers, such that study findings may more readily become actionable science. Findings of this study have implications for effective resource management and may serve as a first step toward integrating FEGS into local NEP planning strategies.

## 2. Methods

### 2.1. Study area

#### 2.1.1. Tillamook Bay, Oregon

Tillamook Bay is located on the Oregon coast, with five rivers (i.e. Kilchis, Miami, Tillamook, Trask and Wilson rivers) converging into the ~34 km^2^ estuary before emptying into the Pacific Ocean (TEP, Tillamook Estuaries Partnership 2019; Appendix A; Table A1). The two largest tributaries are the Trask and Wilson rivers that together form the majority of the Tillamook Bay Basin alluvial plain (Nelson et al. 1998; U.S. Army Corps of Engineers 2005). Tillamook Bay is completely enclosed in a single, mostly rural county with approximately 27,000 residents. The City of Tillamook is located on the broad alluvial plain south of the bay and has been subject to numerous flooding events over several decades (Levesque 2013). Roughly half of the estuarine area is exposed as intertidal mud flats at low tide that can impede navigation to certain areas. However, a deep-draft channel is regularly maintained from the estuary mouth to boat harbors on the north end of Tillamook Bay and accommodates commercial and recreational boating activities (U.S. Army Corps of Engineers 2005). The Tillamook Estuaries Partnership (TEP) was established to build community collaborations to develop and implement plans to manage natural resources of Tillamook Bay and nearby estuaries, including nature-based tourism and recreation (TEP, Tillamook Estuaries Partnership 2019; Appendix A).

#### 2.1.2. Tampa Bay, Florida

Tampa Bay is a much larger estuary (~1,030 km^2^) located on the central Gulf of Mexico coast of peninsular Florida. In stark contrast to Tillamook Bay, Tampa Bay abuts three counties and three major cities (Tampa, Clearwater and St. Petersburg) with a surrounding population of just under three million people (Table A1). Major tributaries into Tampa Bay include the Hillsborough, Alafia, Little Manatee and Manatee rivers, and the larger bay is typically divided into four sub-areas for planning purposes. Old Tampa Bay is in the northwestern quadrant; Hillsborough Bay is in the northeastern quadrant with the river of its namesake; Middle Tampa Bay is the largest central area of the bay; and Lower Tampa Bay is at the mouth. The watershed spans over 2,000 square miles with urban development the predominant land use category (39%), followed by agriculture (20%), wetlands (17%) and other vegetated/forested lands covering approximately 12% (TBEP 2017). As with the TEP, the Tampa Bay Estuary Program (TBEP) was developed to work with community stakeholders to develop and implement plans to manage estuarine natural resources, including recreational uses (TBEP 2017; Appendix A).

### 2.2. Data collection

Similar to other studies involving an analysis of recreational ecosystem services (Dubininkas and Consulting 2016; Levin et al. 2017; Angradi et al. 2018; Oteros-Rozas et al. 2018; Sinclair et al. 2018; van Berkel et al. 2018; Ghermandi and Sinclair 2019), we used publicly available photographs shared through an online social media platform for an initial review of EEPs depicted and their approximate locations within each estuary. Social media images have been broadly linked to cultural, aesthetic and recreational values (Sessions et al. 2016; Fisher et al. 2018). While a viewshed with sunsets or panoramic views of the landscape was one of the more common themes depicted in social media images (Yoshimura and Hiura 2017), we wanted to assess how and whether complementary site features make one location more desirable than another.

For our analysis, we focused on aquatic habitat and designated the NESCS environment types in Tillamook and Tampa Bay estuaries as: 1) Open Water – Near Coastal Marine/Estuarine and 2) Wetlands. We then used the NESCS Plus 3.0 Webtool (EPA 2021) to determine potential beneficiary subclasses linked to these environment types, although we focused on recreational beneficiary subclasses for most analyses. Tabulated results included all potential EEPs associated with the defined Environment Type-Beneficiary pairings. We then ran the InVEST recreation model (developed by the Natural Capital Project, Sharp et al. 2020) to identify hotspots of recreational use based on the density of geo-referenced images on the online Flickr platform (https://www.flickr.com). We characterized the FEGS depicted in individual photographs and compared results with onsite assessments and user observations to facilitate a comprehensive analysis of the relationship between recreational FEGS and estuarine landscape features. We compared results between estuaries and evaluated whether the broader estuarine setting (e.g. surrounding population density, landscape and environmental characteristics, etc.) changed realized recreational benefits, as reflected by the diversity and extent of FEGS. Table 2 outlines each of the data sources, their relevance to the study, the scale of results, and their link to key hypotheses.

#### 2.2.1. Identification of recreational hotspots

InVEST is an online tool based on photo-user-days (PUD), defined as a unique photographer who took at least one photo from a specific location on a - particular day, and predicts recreational use from publicly available, geo-tagged Flickr photographs (Wood et al. 2013; Sharp et al. 2020). We ran basic InVEST models (no predictors) separately for each year (2014–2017). We did not include predictor variables for basic linear regression within InVEST because the model was not designed to discriminate between FEGS (i.e. directly linked to EEPs) and non-FEGS (e.g. selfies, photos inside a food venue, etc.) in assessing recreational use. The model extent was set to the respective watershed boundaries, and we used a hexagonal spatial grid with a 1-km distance between centroids. The model output included ArcGIS (Version 10.2.2) shapefiles and related tables defining the monthly and yearly average PUD per hexagonal grid cell. We performed a cluster analysis (ArcGIS Spatial Analyst) to assess locations within the watershed with the highest average PUD. Grid cells from each watershed that overlapped predominantly coastal and estuarine land cover classes (using the Coastal and Marine Ecological Classification Standard (CMECS, FGDC, Federal Geographic Data Committee 2012) for Tillamook Bay, and the Florida Land Use, Cover, and Forms Classification System (FLUCCS, DOT 1999) for Tampa Bay), were identified as locations likely to facilitate recreational FEGS rather than recreational opportunities reliant on built infrastructure (e.g. stadiums, golf courses, etc.). Lastly, for each year and watershed, we sub-selected the ten grid cells with the highest average PUD for further Flickr image analysis.

#### 2.2.2. Flickr FEGS characterization

As the InVEST model for recreation is based solely on PUD and does not evaluate photo content, we downloaded images directly from the Flickr platform for subsequent analyses. Within the ten grid cells of highest PUD sub-selected in InVEST, we evaluated the subject matter depicted in publicly available images posted to the Flickr photo-sharing platform through the Flickr application programming interface (API). The R packages ‘httr’, ‘RCurl’, ‘rjson’ and ‘XML’ were used to extract images from the Flickr platform (Couture-Beil 2018; Lang 2021a, 2021b; Wickham 2020). The R packages ‘dplyr’, ‘lubridate’ and ‘stringr’ were used to clean and prep data outputs to facilitate subsequent evaluation (Grolemund and Wickham 2011; Wickham 2019; Wickham et al. 2021). We downloaded all photographs and metadata between 2014 and 2017, corresponding with results from the InVEST recreation model. For each year, the API query parameters included: centroid coordinates of the top ten PUD grid cells from InVEST model results, a centroid distance of 0.5 km, and Jan-1/Dec-31 as respective minimum and maximum dates taken. We also limited results to photographs with confirmed geographic coordinates. There was no additional filtering based on image tags or titles. Metadata retained for each image included a photograph identifier, along with the date taken and hyperlink. We then added a location identifier based on the centroid coordinates of the query. We sorted images based on the date, location and photograph identifier. We deleted any duplicate images using the unique photograph identifier. All photos with a working hyperlink were subsequently viewed and categorized.

**Figure 1.**
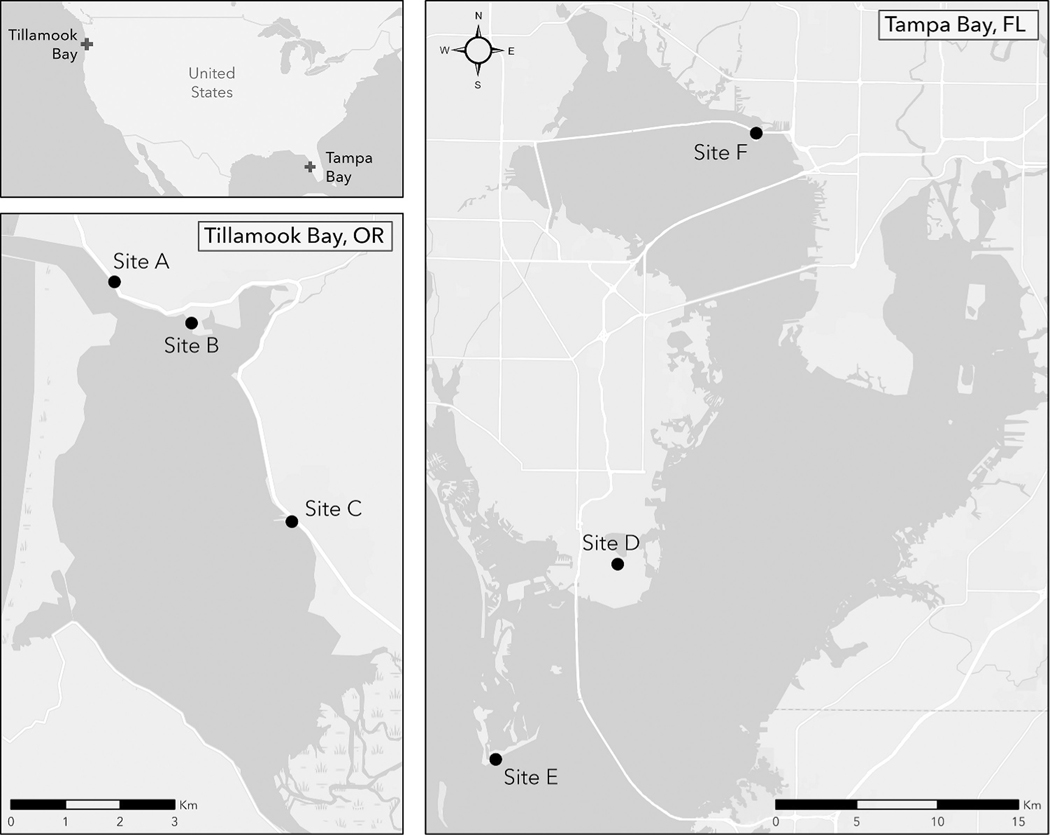
Overview map of study sites (i.e. A–F) in both Tillamook Bay, Oregon and Tampa Bay, Florida. Tampa Bay is roughly 30 times larger than Tillamook Bay, as clearly shown by the different magnitudes of the scale bar insets in the respective maps.

Images were manually classified based on the main subject of the photograph. We modified the approach of Angradi et al. (2018), which used a subject matter characterization largely based on the FEGS-CS (Landers and Nahlik 2013), into a revised list of features that were then sorted into the most appropriate EEP subcategory per NESCS (Table 3). FEGS require beneficiaries (Newcomer-Johnson et al. 2020), thus, images with people engaged in activities where the link to the ecosystem was not readily apparent or direct were classified as non-FEGS and excluded from further analysis. In instances where several photographs of the same subject matter were taken by a single owner on the same day, the PUD was only counted once in subsequent statistical analyses. For example, a birder might take several bird photos on a single outing, or someone recreating on the beach could take several photographs of the area from different angles. In these cases, we counted each photographer as having posted just one image of that subject matter. This step was necessary to avoid arbitrarily over-inflating the importance of any associated EEP or beneficiary subclass based on an individual’s decision to capture multiple images of the same FEGS. In contrast, pictures taken by a single photographer on the same day, but depicting different subject matter were included. Consistent with PUD estimates, a picture taken by the same photographer of the same subject, but on a different day, was also included. Lastly, we subset Flickr images from the three highest PUD grid cells within each estuary identified previously by the InVEST models that also overlapped natural landscape features. These three cells in each estuary (i.e. Tillamook Bay or Tampa Bay) were selected as study sites (for a total of six sites), which were arbitrarily assigned site designations A-F (Figure 1). The final Flickr dataset consisted of the summation of PUD by estuary, year, month, site and EEP. The site variable was not relevant for the Flickr image analysis but did inform the location of empirical observations.

#### 2.2.3. Site-station assessments

**Figure 2.**
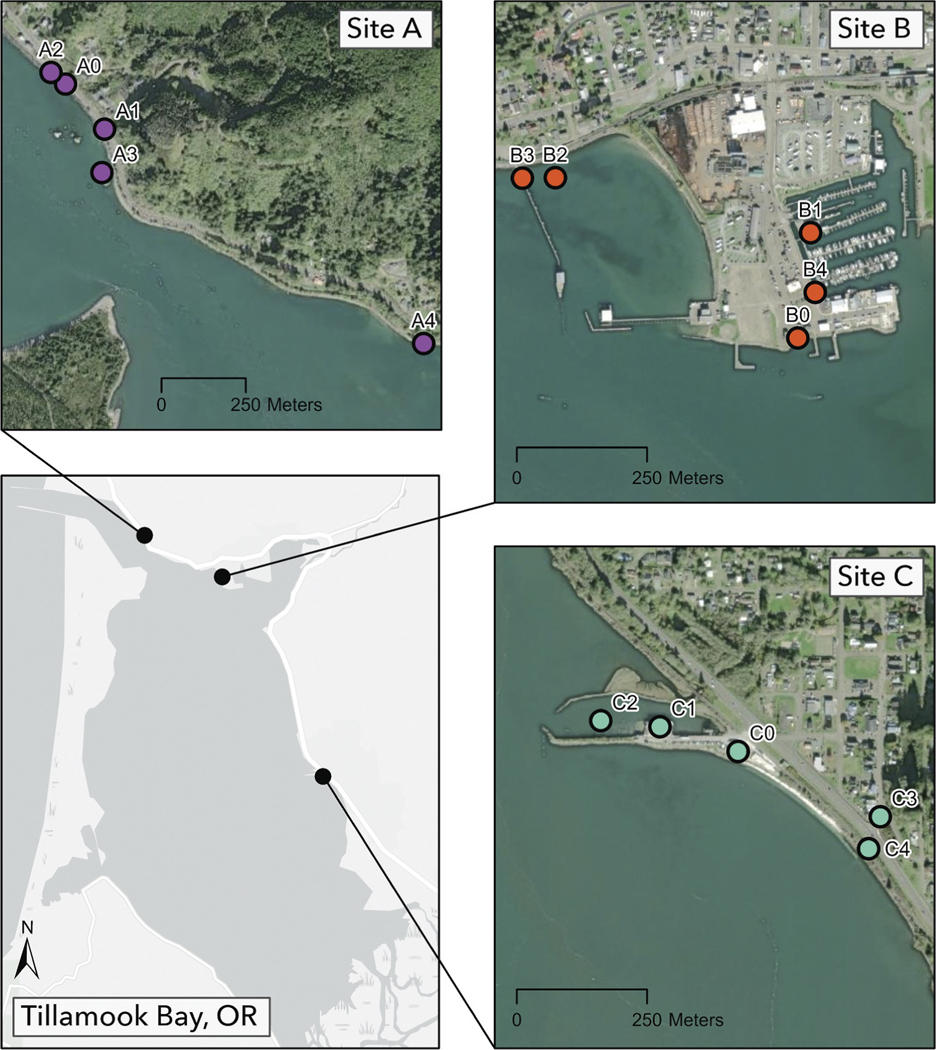
Map of survey stations nested within Tillamook Bay estuary sites A–C.

**Figure 3.**
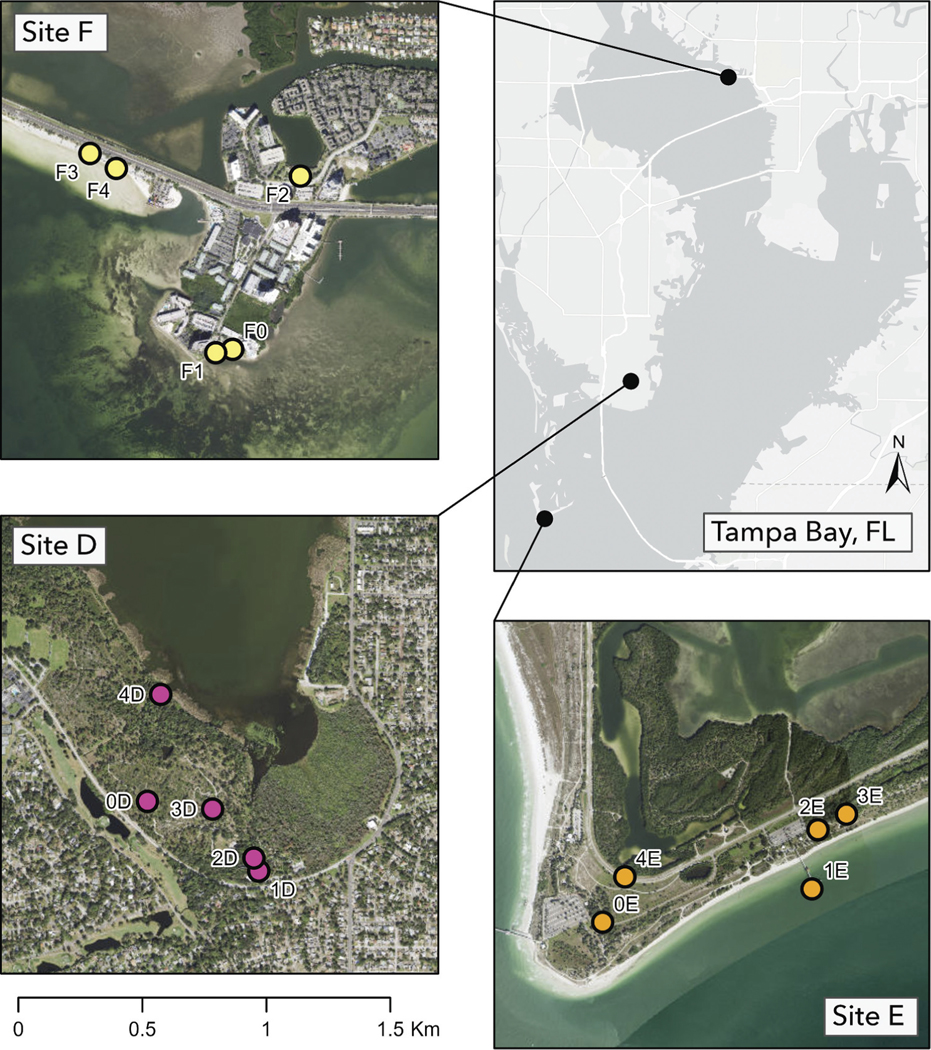
Map of survey stations nested within Tampa Bay estuary sites D–F.

We conducted onsite assessments of the landscape features present at the selected study sites. Within each study site, five geotagged image locations were randomly selected via ArcGIS to sample as stations for field surveys. We limited the selection process to unique photographers and coordinates ≥50 m apart from one another. The latter condition helped ensure that the five sampling stations within each study site captured a range of natural and human-made attributes present within a site of an approximate 0.5-km radius (Figures 2 and 3).

Some attributes (e.g. roadways, the viewscape, birds, etc.) were observed across stations, and this was expected. This consideration of finer-scale, natural and human-made attributes (i.e. beyond the mapped land cover class) allowed us to decipher how different places might support or encourage recreational FEGS use and whether utility changes at the station or site level.

**Figure 4.**
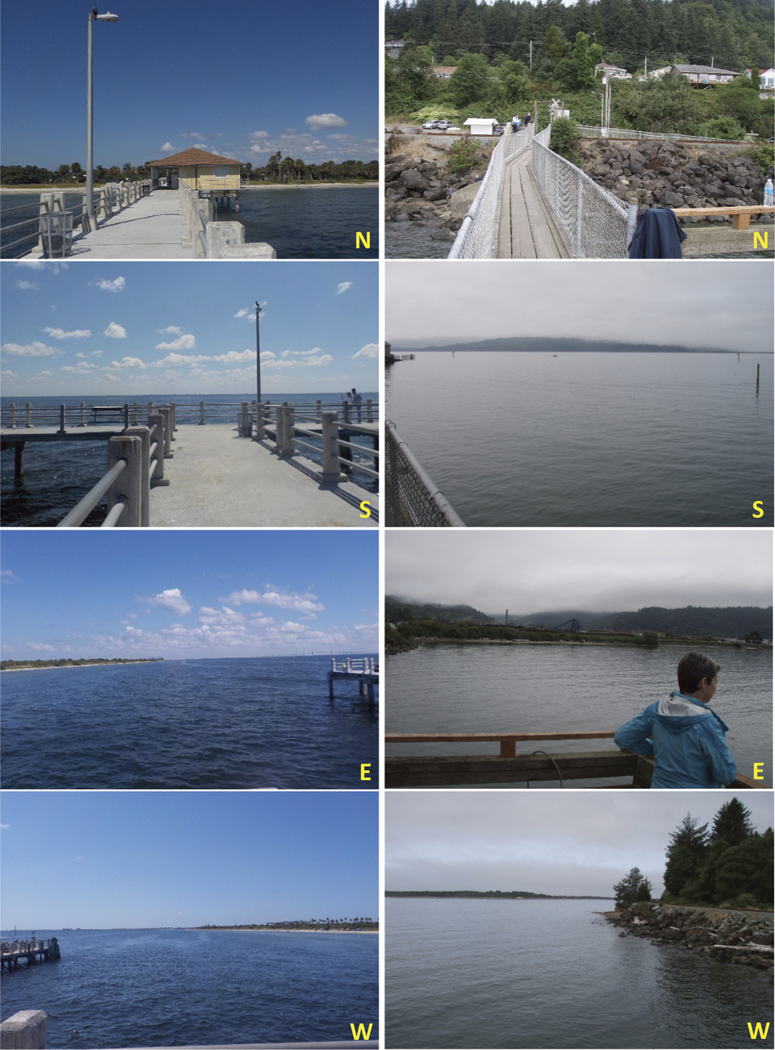
Photographs taken during onsite assessments at two stations from different estuaries but within the same station group Beta. The station groupings were based on the degree of similarity in the natural and human-made attributes present. Left panel photographs are from Tampa station E1; right panel photographs were taken at Tillamook station B3.

We used a handheld GPS unit (Trimble GeoExplorer 6000) to navigate to each station where we captured four pictures, one in each of the cardinal directions (N, S, E, W), to establish the 360-degree viewshed available at each station. Lastly, we documented the natural and human-made attributes present at each location that could facilitate FEGS availability (Figure 4).

#### 2.2.4. Direct observations of people engaged in recreation

We recognize that Flickr, as with any other online platform for sharing information, is biased toward the people choosing to use this platform and against those using alternate photo-sharing platforms, or those who do not post images to social-media websites. There may also be photo biases across beneficiary groups and decisions about how and where to share images. It is unclear to what extent the subset of Flickr users reflected all potential recreational beneficiaries within an estuary. We used NESCS recreational beneficiary subclasses to characterize individuals that were passively encountered (Table 4). In August 2018 (Tillamook Bay) and February 2019 (Tampa Bay), we conducted passive onsite observations (i.e. without initiating direct contact or otherwise engaging the people present) at each station to directly sample recreational beneficiaries of each estuary. While we were initially targeting sampling periods corresponding with months with higher PUD detected in the Flickr dataset, the final sampling dates were constrained by surveyor availability and logistics.

Prior to conducting the surveys, a two-hour preliminary field observation was conducted at a randomly selected sampling station within each of the three sites in Tillamook Bay to determine the optimal time window to observe station users and the most efficient amount of time to survey (or observe station users) at each station. We navigated to each station and established a 25-m radius, considered the effective area from which to accurately observe people recreating there. We documented the abundance of people engaged in various recreational activities (i.e. recreational beneficiaries) and subsequently classified people at each station based on the NESCS recreational beneficiary subclasses listed in undefined 2. We determined that 20 min was a sufficient sampling time per station to document representative recreational use within a given two-hour period; however, observed counts were still highly variable (Appendix B). With regard to scenic viewers, we devised a sampling protocol to count passers-by as active scenic viewers only if they paused to observe (for ≥10 s), point at, or take a picture of the viewscape or natural feature.

Following preliminary time trials, we conducted three surveys of people’s activities at each station (for a total of 15 surveys per site); each station survey occurred on a different day and at a different time within the ‘optimal’ time window in order to capture a wide spectrum of activity. The sampling order of stations within a site was randomly established. Upon arrival to a station, two observers selected a vantage point with an unobstructed view of the station that did not directly influence other people’s access to or potential use of FEGS. On individual datasheets, observers recorded the number of people recreating and the subcategories of FEGS utilized, along with ancillary data about the survey (e.g. time, weather conditions and temperature). Weather was characterized as sunny, cloudy, overcast, foggy, or rainy, as defined by the World Meteorological Organization (https://public.wmo.int/en). Temperature was recorded at the beginning of each survey.

### 2.3. Statistical analyses

All results were evaluated using R statistical software (R version 4.3.1, R Core Team, 2023). We grouped Flickr images by NESCS beneficiary classes (i.e. Commercial/Military Transportation, Commercial/ Industrial, Government/Municipal/Residential and Recreational). However, since the majority of images reflected recreational beneficiaries, subsequent statistical analyses focused solely on differences between *apparent* subclasses of recreational beneficiaries. Assumptions of normality and homogeneity of variance were evaluated using the Shapiro-Wilk and Bartlett Tests, respectively (R package ‘stats’). When those assumptions were not met, a nonparametric Kruskal-Wallis Rank Sum test was used to test for differences in PUD by recreational beneficiary subclass and natural EEP attribute. A follow-up Dwass-Steel-Crichtlow-Fligner test was run for pairwise comparisons of recreational beneficiary subclasses with unequal variance. We created an alluvial diagram (R package ‘ggalluvial’, Brunson and Read 2023) to visualize the amount of PUD evidence linking apparent recreational beneficiary subclasses in each estuary to EEPs.

We tested for potential spatial autocorrelation in the total users observed at each station using Moran’s I test statistic (‘Moran.I’ function in R package ‘ape’, Paradis et al. 2004) and the inverse distance spatial weights matrix. We also ran the test in ArcGIS (Spatial Autocorrelation – Global Moran’s I) to validate results and generate a summary report. Station attributes were then evaluated to understand how and whether the presence of shared attributes across stations may have shaped perceived FEGS availability, and the subsequent number of users observed. Within each estuary, results from onsite assessments formed a matrix with rows corresponding to the five stations within each of the three sampling sites (15 stations per estuary) and columns corresponding to the suite of potential natural and human-made attributes (see Table 1). Within the matrix, there was either a 1 or 0 entry noting the presence or absence, respectively, of attributes at a station. Columns with all zero entries (i.e. attribute(s) missing from all stations) were removed prior to analysis. Nonmetric multi-dimensional scaling (NMDS) was then used to reduce station attribute data into two dimensions and make comparisons across sampling stations. With no assumptions of linearity, the NMDS arranges points to maximize rank-order correlation between the actual distance and ordination space distance through an iterative process. We used an underlying Bray-Curtis dissimilarity index corrected for binary data and evaluated goodness-of-fit based on the stress value and Shepard diagram plotting ordination distance against observed dissimilarities (‘metaMDS’ function in R package ‘vegan’, Oksanen et al. 2020). The ‘metaMDS’ function in R performs a principal component analysis (PCA) on the ordination results, so axis-1 of the NMDS plot will explain a greater proportion of the overall variance. As with other ordination techniques, points closer together are more similar than those farther apart. Lastly, we ran a *k*-means clustering algorithm to determine the optimal number of station groups (*k*) to minimize within-cluster variability. We tested *k* = 1 up to 14 station groups and looked at the scree plot to determine the number of groups to retain based on the reduction in within-group error. We then plotted those groups in ordination space to visualize station clusters in multidimensional space associated with natural and human-made attributes (R packages ‘ggplot2’ and ‘ggrepel’, Wickham 2016; Slowikowski 2021).

With a new potential grouping variable for stations with similar attributes, we ran regression models to determine which explanatory variables were significant predictors for the number of observed users. We ran zero-inflated negative binomial (ZINB) mixed-effects models (R package ‘glmmTMB’, Brooks et al. 2017) to test the main effects of site, and station group (i.e. groups of stations with similar natural and human-made attributes and subsequent *k*-means results discussed earlier in methods) for significance in explaining variance in the total number of recreational beneficiaries observed. Zero-inflation was added assuming an intercept-only model and conditioned on estuary or time of day. Estuary (Tillamook or Tampa bays) was added as a random effect in recognition that each estuary has a suite of other characteristics that were not explicitly included in the model. Additionally, our study was limited to a sample of two estuaries out of all possible estuaries and treating this variable as a random effect allows us to test for differences in observations due to main effects reflecting onsite conditions dictating the recreational experience. Weather (i.e. sunny, overcast, foggy) and time of day (i.e. morning or afternoon) were tested as both fixed and random effects in models, with their ultimate inclusion or exclusion determined by the proportion of variance explained and overall model fit. We ran a global model with all potential explanatory variables, then generated a model selection table with model subsets (‘dredge’ function in R package ‘MuMIn’, Barton 2020) for comparison. We then viewed the quantile-quantile and residual plots to further assess fit of the models with the lowest AICc. R package ‘sjPlot’ was used to visualize ZINB model coefficients and marginal effects (Lüdecke 2021). Assumptions of homoscedasticity were confirmed based on the residual plots (R package ‘DHARMa’, Hartig 2021). We calculated the estimated marginal means for each significant predictor, with results averaged over the levels of other variables and a Tukey p-value adjustment to account for pairwise comparisons, to determine which factor levels differed for variables included in the best performing models (Lenth 2021).

## 3. Results

### 3.1. Flickr FEGS characterization

**Figure 5.**
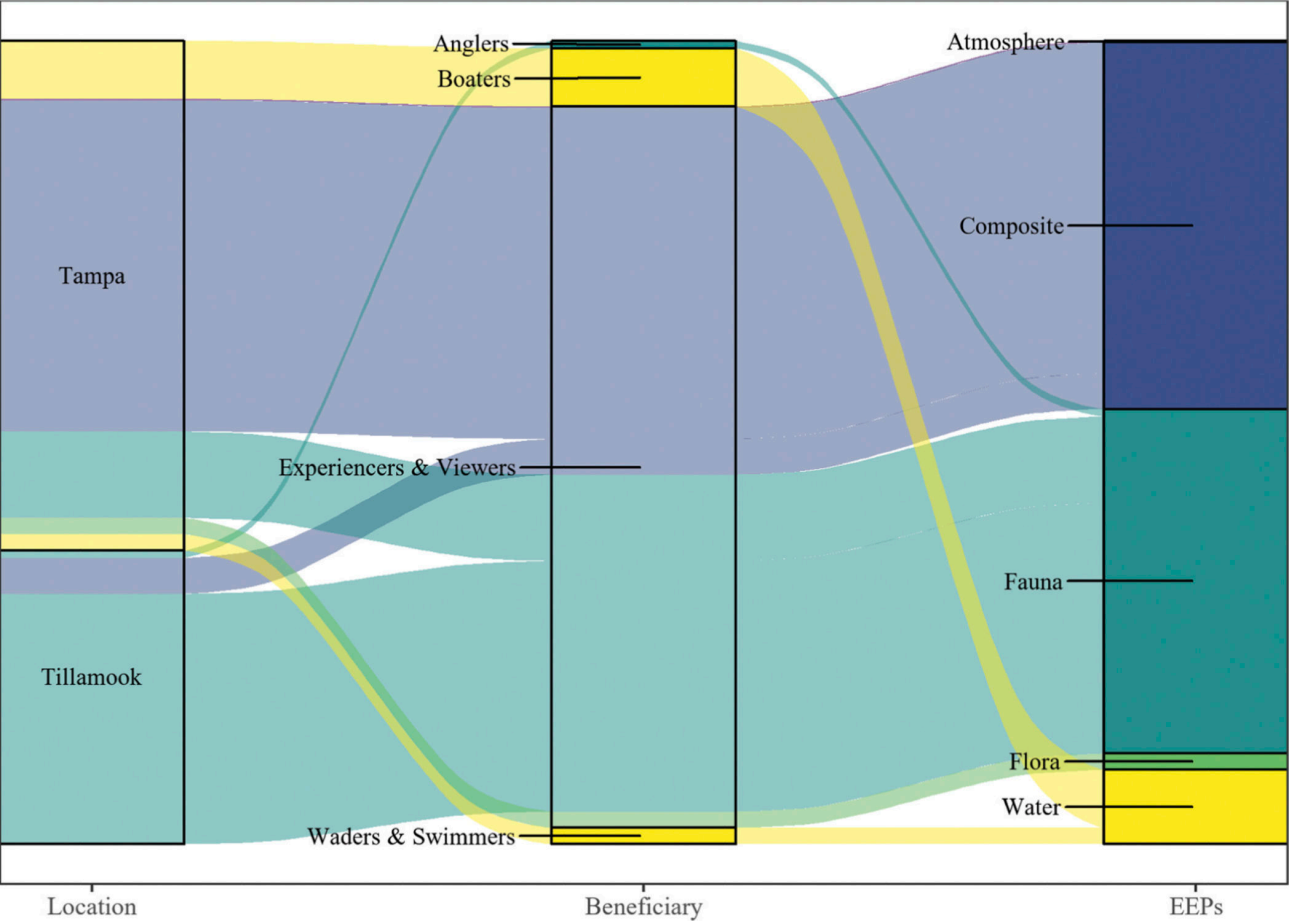
Alluvial diagram with the width of ribbons indicating the number of photo-user-days (PUDs) linking the apparent recreational beneficiary subclasses from each estuary on the left to the ecological end products (EEPs) on the right. NESCS recreational beneficiary subclasses included ‘experiencers and viewers’; waders and swimmers; boaters; and anglers observed in either Tillamook Bay or Tampa Bay. EEP categories reflected in images included composite features, atmosphere, fauna, flora and water. The ribbon colors correspond to the EEP categories.

A total of 1,384 Flickr photos were individually evaluated and characterized based on image content. Most photos reflected non-FEGS (i.e. 51% in Tillamook Bay and 59% in Tampa Bay). However, recreationalists were the most prevalent NESCS beneficiary class detected in the remaining images, reflected in 45% of the images from Tillamook Bay and 36% of the images from Tampa Bay. Other NESCS beneficiary classes were reflected in 3% or less of the images in either bay (Table 5). Most PUD in Tillamook Bay were associated with fauna, whereas composite features were more common in PUD from Tampa Bay (Figure 5). Water and fauna were the other two EEP categories most often featured in Tampa Flickr photos (Figure 5). Recreational ‘experiencers and viewers’ was the most dominant recreational beneficiary subclass, and the majority of photos associated with them depicted fauna in Tillamook Bay and composite features in Tampa Bay (Figure 5).

### 3.2. Site-station assessments

**Figure 6.**
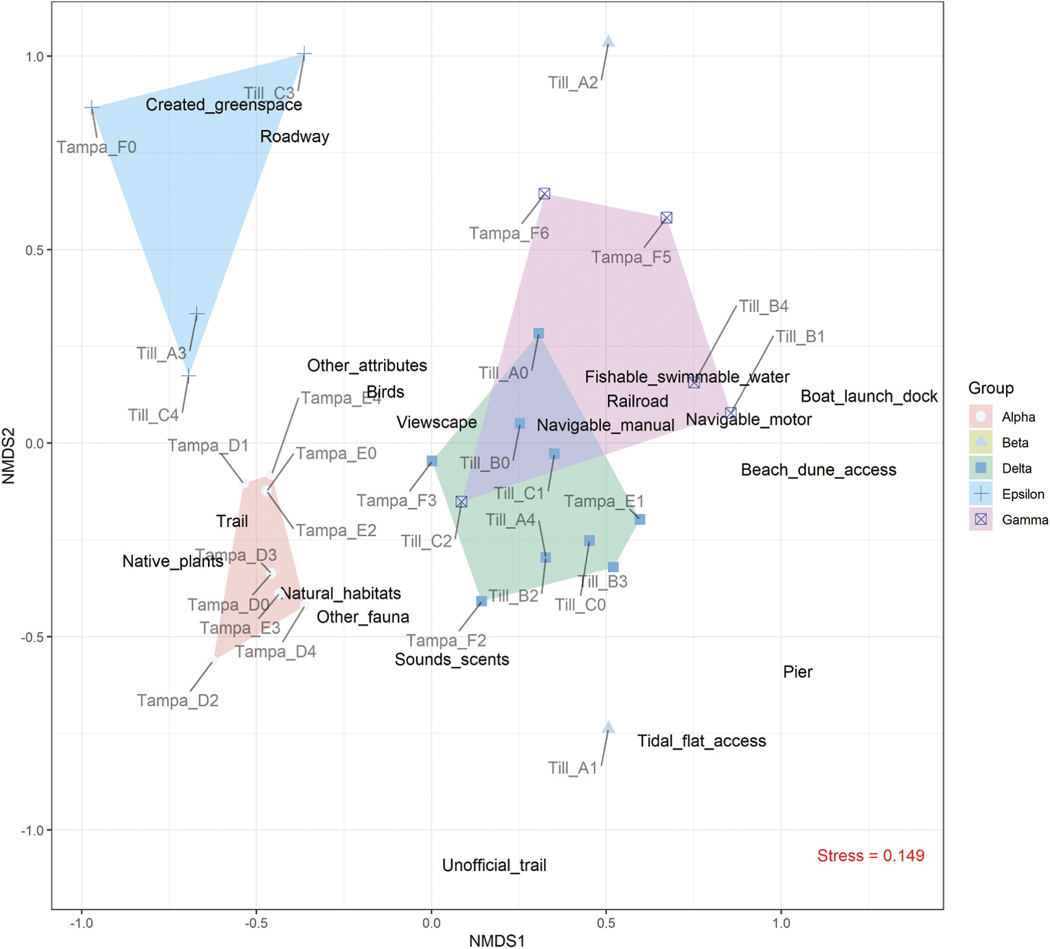
Nonmetric multidimensional scaling (NMDS) results showing similarity between stations and observed attributes in ordination space and clusters of similar stations. Station groups were designated by Greek letters, as shown in the legend, with polygons in the figure corresponding to their alignment in ordination space along with distinguishing site features. Individual stations are plotted with labels depicting the location (i.e. ‘till’ for Tillamook Bay and ‘Tampa’ for Tampa Bay), site and station (e.g. Till_A1 denotes Tillamook Bay, site A, station #1). Station groups are represented by distinct colors and symbology within the plot, as indicated by the legend key to the right of the plot.

Moran’s I test result indicated no evidence for spatial autocorrelation (clustering or dispersion) among stations (Appendix C). Ordination revealed differences among stations as a function of their environmental attributes (Figure 6). There was agreement between the Bray-Curtis dissimilarity rank-order and the Euclidean distance measured in ordination space, with an approximate stress value of 0.15 and non-metric *R*^*2*^ of 0.978. Water-related natural and human-made attributes (e.g. navigable water, fishable/swimmable water, boat launch or dock, and beach/dune access) were close together in ordination space and typically farther away from stations with trails, plants, other natural habitats, and fauna other than birds (Figure 6). When plotting stations in ordination space, some were more or less aligned with these observed attributes than others. The final station groups reflected stations with greatest similarity within this ordination space. Station Group Alpha was most aligned with trails, native plants and natural habitats; Station Group Beta was aligned primarily with viewscapes (see Figure 4 for an example of cross-estuary station similarities); Station Group Gamma was aligned with greenspace and proximal roadways; Station Group Delta appeared to contain the outlier stations (i.e. A1 in Tillamook Bay and D2 in Tampa Bay), one of which had tidal flat access; and Station Group Epsilon was aligned with navigable, fishable and swimmable waters, along with viewscapes and the presence of a railroad (Figure 6). Results are presented in tabular form to clearly show natural and human-made site features common to each station group (Table 6).

### 3.3. Direct observations of people engaged in recreation

**Figure 7.**
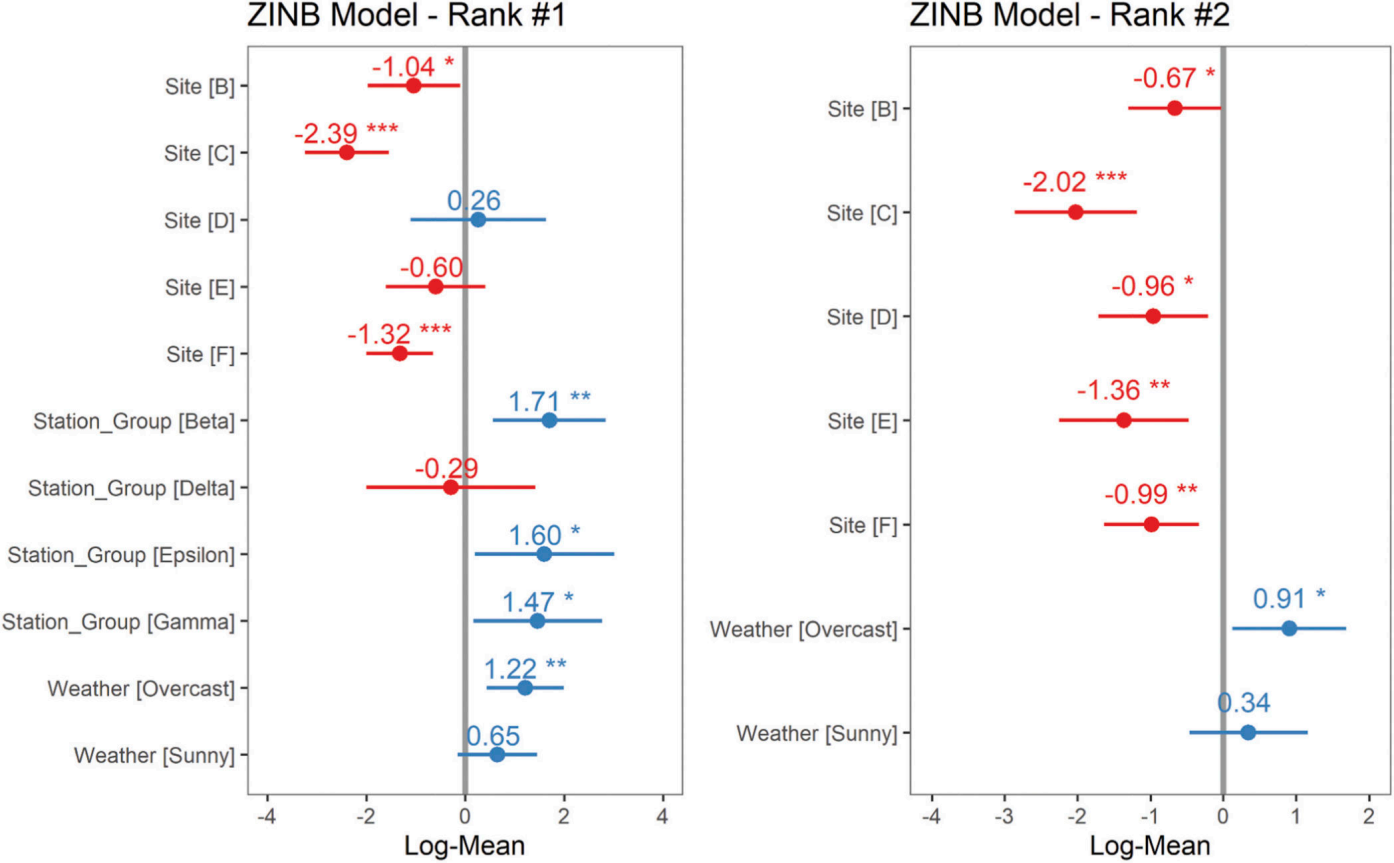
Model coefficients (untransformed) for the top two models predicting the total number of recreational beneficiaries observed. Error bars intersecting the ‘neutral line’ at 0 indicate no effect (i.e. no significant difference between the number of recreational beneficiaries relative to the reference site (Tillamook site A), station group (Alpha) (i.e. in the model including station as a factor), or weather condition (foggy)). In the top ranked model including the station variable, station group Alpha (i.e. the stations most aligned with trails, native plants and natural habitats) had significantly fewer recreational beneficiaries than station groups Beta, Epsilon and Gamma. See Figure 3 for the full suite of natural and human-made attributes mapped in ordination space with station groups. Both of the models indicated most sites had significantly fewer recreational beneficiaries than Tillamook site A.

Site, station group and weather variables were retained in the two top models for the predicted number of recreational beneficiaries (Table 7). Each model included a random effect for estuary and an intercept-only model for zero inflation, which assumes a constant process leading to excessive zero counts (Figure 7). Post-hoc comparisons of sites, holding all other variables in the model at their average, indicated Tillamook Site A had significantly more recreational users than most other sites. All sites within Tillamook Bay were on the perimeter of the bay and within a few hundred meters of a major interstate highway and there were several passive scenic viewers taking photographs while in route. In addition, a railroad with a scenic passenger train offered daily tours during the survey period and passed through Tillamook Bay Sites A and B, with each train carrying up to 100 passengers. This likely resulted in the higher count of recreational beneficiaries in Tillamook Site A. In contrast, two of the three Tampa Bay sampling sites were inside state or locally designated natural areas, with entrances (and fees). The final site on the northern edge of Tampa Bay (i.e. Site F) was adjacent to a major freeway, but with significantly more traffic and higher speeds such that passive users in cars were not easily observable.

When looking across the stations that seemed to share similar natural and human-made attributes, significantly more people were present at Station Group Beta than at Station Groups Alpha (*p* < 0.05) or Delta (*p* < 0.05). Station Group Beta was most prominently associated with viewscapes. Weather also factored into the predicted number of recreational beneficiaries, with fewer people observed on foggy days compared to days with overcast (*p* = 0.01). This may further highlight the relative importance of the viewscape to the activities that people were engaged in, as foggy conditions often impede visibility. In contrast to PUD results from Flickr images, we observed more people recreating in Tillamook Bay (*n* = 712) than Tampa Bay (*n* = 434). However, the standard deviation of the estuary term in both of the top two mixed-effects models was <2.5 E-5, several magnitudes lower than the absolute value of the coefficients for any of the significant fixed effects (Figure 7). Results indicate that there was not a distinguishable difference in the number of recreational beneficiaries in each estuary, after accounting for variance due to the finer-scale site and station elements that appeared more relevant for predicting the number of people recreating.

While recreational beneficiary subclasses were not exclusive (i.e. a person could be both angling and taking photographs), ‘experiencers and viewers’ were the most prominent recreational beneficiaries in both estuaries, representing over 98% of the beneficiaries observed. This was consistent with results from the PUD analysis (Figure 5). Given the overwhelming dominance of recreational ‘experiencers and viewers’, we did not test separate models for each recreational beneficiary subclass.

## 4. Discussion

### 4.1. Revisiting study hypotheses

General consensus has been reported on the value of coastal areas to recreational and cultural ecosystem services (Granek et al. 2010; Loomis and Paterson 2014; Yoshimura and Hiura 2017), but not necessarily on how those values may change across the landscape or between estuaries. This study demonstrates how EEPs contribute realized benefits to people recreating in coastal estuaries, as indicated through both revealed preference (i.e. Flickr images) and passive observation of people’s onsite activities. We revisit the main hypotheses and summarize key findings below:

**H**_**0**_**1:** Flickr images will show a greater diversity of recreational users in Tampa Bay than Tillamook Bay.

**Fail to Reject** – As shown in Figure 5, there were more recreational subgroups reflected in photos taken in Tampa Bay compared with Tillamook Bay.

**H**_**0**_**2:** Dominant recreational beneficiaries will differ between Tampa and Tillamook bays.

**Reject** – ‘Experiencers and viewers’ were the dominant recreational subgroup detected in both estuaries.

**H**_**0**_**3:** Tampa Bay will have more recreational users observed than Tillamook Bay, but the diversity of recreational beneficiaries will be comparable.

**Reject** – There were more recreational users observed in Tillamook Bay. Both estuaries had two recreational beneficiary subgroups observed, although ‘experiencers and viewers’ were the most prevalent.

**H**_**0**_**4:** Location (sites and stations) will account for significant variability in the number and suite of recreational beneficiaries.

**Fail to reject** – Sites and station groups based on common attributes present were indeed significant predictors for the number of users observed. However, there was insufficient data to detect potential differences in recreational beneficiary subclasses.

**H**_**0**_**5:** Stations with more attributes will correspond with greater numbers of recreational users and beneficiaries.

**Reject** – Station Group Beta was primarily aligned with the presence of a viewscape, yet it was a significant, positive predictor for the number of observed users. Station Group Epsilon was also a significant positive predictor and was associated with four site attributes. Thus, the type of attribute(s) may be more important than the absolute number when predicting recreational utilization.

**H**_**0**_**6:** Stations with similar attributes will have a similar portfolio of recreational users.

**Insufficient information** – Sampling was biased toward recreational ‘experiencers and viewers’ but there was not enough data to evaluate the full portfolio of potential recreational subgroups.

**H**_**0**_**7:** Onsite user observations will capture a greater diversity of recreational beneficiary subclasses than Flickr data.

**Reject** – Flickr images reflected more recreational beneficiary subgroups than onsite observations.

### 4.2. Common recreational values and beneficiaries in coastal estuaries

Recreational users of Tillamook Bay and Tampa Bay were remarkably similar, despite the considerable difference in size, climate and geographic location. After accounting for finer site-scale natural and human-made attributes, essentially no difference was found in the portfolio of recreational beneficiary subclasses, associated EEPs, or the intensity of apparent FEGS utilization. The higher absolute number of people observed recreating in Tillamook Bay in comparison with Tampa Bay likely reflected the fact that recreational users in Tampa Bay are dispersed over a much larger area and the probability of observing multiple users at the scale of a single station is simply lower. In contrast, the greater levels of PUD recorded in Tampa likely reflected the larger surrounding population and greater numbers of people utilizing the Flickr platform in the region. Seasonality likely affected onsite survey results and PUD. Tampa Bay is in Florida and has a much wider tourism season, but surveys were conducted in February and during the week when the number of recreational users may have been lower. In comparison, Tillamook surveys were conducted in August during peak summer tourism on the Oregon coast. Recreational ‘experiencers and viewers’ were prominent in both estuaries and the evaluation of their distribution and interaction with the environment through different data lenses provided a holistic view of attributes such as water views, pedestrian access and the presence of birds and other fauna that appear to coincide with continued utilization.

The present study highlighted coastal recreational beneficiary subgroups and linkages to EEP as reflected in data from more passive users that could be overlooked in traditional FEGS assessments, with an admitted bias toward land or shoreline-based activities. We began at the landscape scale to hone-in on estuarine land cover classes, but delved into characteristics of each station to understand how people seeking recreation might derive value from a particular location. Grêt-Regamey et al. (2015) conducted a meta-analysis of recreational ecosystem services literature and found four types of information commonly used to assess service provision: land use; human access infrastructure; valuation; and landscape aesthetics. They noted differences in the type of data needed based on the scope (i.e. tier) of the question or problem (Grêt-Regamey et al. 2015), and this approach seems helpful in the context of adaptive coastal resource management. In our study, we integrated datasets with information pertaining to land use, access features like trails and roadways, and aesthetics; however, we did not explicitly include valuation. The methodology used in our study did not necessarily allow for accurate valuation because a spatial discrepancy may exist between photographers and content in geo-referenced photos assumed to reflect revealed preference values (Byczek et al. 2018) because photos can be taken from quite a distance and values reflected in images may not actually be present where the photographer is standing. Additionally, assigning explicit values based on image content, without supplemental data from the photographers themselves, would have likely introduced more bias and assumptions that may or may not have accurately reflected the perspective of individual photographers. A study explicitly designed to understand how images relate to an individual’s willingness to pay for certain features would likely need to link complementary survey responses from the same individuals taking photographs to enable more accurate valuation. Still, the content in Flickr images reflected many features that were corroborated during direct onsite observations. The composite EEP category, which includes a compilation of natural attributes and the viewscape, was commonly featured in Flickr images and similarly reflected in the station grouping variable included in the best model predicting the number of people recreating. The larger estuary setting (e.g. Tillamook and Tampa bays) explained very little of the variance in the number of people recreating and stations with more similar attributes were not necessarily within the same estuary. Natural composite attributes and access points at the site and station scale were most important to observed recreational beneficiaries.

### 4.3. Viewing recreational use through multiple data lenses

Images posted to social media can often complement more traditional survey data (Sessions et al. 2016; Fisher et al. 2018; Hausmann et al. 2018), and we similarly found that higher-use sites identified by the InVEST recreation model (i.e. based on the density of Flickr images) coincided with a greater number of people observed recreating during onsite surveys, especially recreational ‘experiencers and viewers’. Photo evaluation and classification required sifting through a profusion of other data. Publicly available photo-sharing platforms were not designed for the purpose for which we have used them, and it is not surprising that a large percentage of photos did not depict nature-based recreation or EEPs. Whereas the majority of photos encountered on the Flickr platform did reflect recreational activities, many were associated with built infrastructure rather than natural features on the landscape (e.g. sporting events in an arena, amusement parks, commercial tourist attractions, etc.). Even after filtering InVEST model results to focus on image locations overlapping water and wetland landcover classes, a relatively high volume of photos depicted non-FEGS. This does not negate the utility of the InVEST recreational model in helping assess the spatial distribution of recreational users because a positive relationship generally exists between the number of geotagged images and independent surveys of people at a site (Wood et al. 2013). Nonetheless, results do highlight the need for additional information when attempting to understand exactly how users are deriving value from the landscape. Van Berkel et al. (2018) paired photo records with a high-resolution, LiDAR-based digital surface model to construct individual viewsheds for each georeferenced location to help answer this question. Similar to other studies (Cunha et al. 2018; Mulvaney et al. 2020), we used empirical evidence from the local area (e.g. the user observations in this study) to enhance our understanding of recreational FEGS that may be available at a particular location.

In studies where tourist destinations such as national parks were the focus, Flickr images may have been more likely to depict recreational FEGS associated with the landscape and mirror observations made through direct survey. In those cases, a positive correlation existed between independent visitor data and social media images posted online (Wood et al. 2013; Sessions et al. 2016; Fisher et al. 2018; Hausmann et al. 2018). In this study, some differences in the EEP categories were apparent in photographs compared with those noted through direct observations of people’s activities. While fauna was a prominent EEP category in photographs (especially from Tillamook Bay), the composite EEP features were most commonly linked to onsite recreational beneficiaries. Some of these differences could be attributed to biases inherent to the methodology, while others could represent differences in the scope of people sampled. For example, the onsite assessments in this study were conducted from the shoreline or surrounding uplands and did not account for people that may have been swimming or engaged in other water-based activities away from the shore. Additionally, Flickr users represent only a fraction of potential people recreating at a location, and Flickr images featuring birds could suggest an affinity for birders to share photography using this particular platform. Some FEGS may also be more amenable to photography than others. Beach-goers planning to enter the water and those recreating on the water may be more apprehensive of taking photographs near water that could damage camera equipment. Similarly, those biking, crabbing from a pier, kayaking and doing other hands-on activities may be less likely to take photographs while engaged in those activities. Enjoying scenic views is passive and not only valued as a FEGS in its own right but easily layered with other recreational activities.

Understanding that recreational beneficiaries make a series of choices at multiple scales before arriving at a given location is important. Recognizing the limitations and scope of available data are equally important. For instance, geo-referenced photo points may only be able to identify broader trends in ecosystem service provision at the landscape scale; whereas looking at the content of photos adds another layer of specificity that can reveal which natural and human-made features were valued. Similarly, while it is widely recognized that aesthetic views are highly favored by people recreating, this information alone may not be helpful for prioritizing coastal site restoration since even impaired waters can offer nice views. In that case, resource managers may be more interested in identifying sites where people are entering the water. We also recognize that not all FEGS are mutually exclusive and many of the locations in this study could accommodate various beneficiaries. We used the NESCS FEGS classification scheme which links human beneficiaries to EEPs and is thus primarily focused on underlying instrumental values, or nature as a resource and asset to people (IPBES 2023). The locations assessed in this study likely included intrinsic and relational values that were not explicitly addressed in this study, though some of the EEP attributes observed indirectly point towards some of the intrinsic values present. There are a broader suite of values and valuation methods that are relevant and merit further consideration as we contemplate the importance of coastal estuaries.

### 4.4. Socioeconomic considerations

Though this study was not designed to fully evaluate potential biases when considering whether there are equal opportunities for individuals to reach a given location per se, or test hypotheses about potential socio-economic barriers to coastal FEGS utilization, results did highlight opportunities for improvement. We did not select tourist destinations *a priori* in this study; nonetheless, the point of access can affect the degree and type of recreational use (Mulvaney et al. 2020). Two public sites facilitating nature-based tourism near Tampa Bay (i.e. Site D in Boyd Hill Nature Preserve and Site E in Fort Desoto State Park) were identified for onsite assessments solely based on the overlay of InVEST model results (which reflects the popularity of those locations) and the presence of wetland/water features. Another station surveyed was in Site F at Ben T. Davis Beach which also provided access to a public beach in an area surrounded by commercial and private property. All three of these locations had nominal costs associated with access (i.e. parking, admission, or entrance fees) that could preclude some people from visiting those places. This potential barrier to everyone enjoying estuarine and coastal FEGS in a large, urban watershed has implications for equitable distribution of FEGS to peoples within Tampa Bay watershed that warrant further study. Similarly, the privately owned lands surrounding much of the Tampa Bay shoreline, necessitating that one sampling location be moved, has significant implications for fair and equitable access to coastal FEGS. Tillamook Bay is also surrounded by privately owned land; it is essential that public access points are both safe and accessible to a diverse group of users. In addition to protecting and enhancing the condition of EEPs sustaining recreation use, resource managers might consider improving equitable access.

Potential inequities in coastal FEGS utilization have not been extensively studied, though the body of research on equitable access to ecosystem services in urban areas is growing (McPhearson et al. 2014; Phelan et al. 2020). Research on the disparities in recreational ecosystem service provision in urban landscapes is fairly recent and primarily focused on greenspaces (Kosanic and Petzold 2020), which may or may not include FEGS associated with natural coastal landscape features. Many questions remain about the optimal management of coastal landscapes to ensure sustained and equitable access to a broad suite of recreational FEGS. Understanding potential barriers to recreational FEGS utilization warrants future study and a broader conversation among coastal communities, land managers and resource agencies.

### 4.5. Proactive management to facilitate sustainable recreational use of coastal estuaries

Even in what some might consider more homogenous forested landscapes, recreational visitation has been positively correlated with trail density (Sonter et al. 2016). These types of access features are recognized as making recreational sites more attractive to potential visitors and stack atop other values (Byczek et al. 2018), and this trend held true for both estuaries. In Tillamook Bay, some people were observed crossing a major highway on foot and walking along railroad tracks to access popular locations with bird colonies or a rocky intertidal beach. This is an issue where resource managers may work with the city, county and the local community to implement intervention strategies that could include signage, outreach, road crossings, or new trails to facilitate safer access to FEGS component features. If so, this would exemplify the suggested tiered approach to adaptive management whereby information about the location and intensity of recreational FEGS use can be integrated into planning frameworks and lead to positive outcomes (e.g. safer access to recreational FEGS) in the community (Grêt-Regamey et al. 2015). Our goal was to not only build on our understanding of how coastal estuaries deliver (or facilitate) recreational FEGS but also to provide estuary managers with relevant information on the location and diversity of recreational beneficiaries that may aid in long-term planning. Decision makers may consider using the types of data collected in this study to help prioritize where to place more access features and reveal which users to target for certain kinds of outreach. Managers may also use this kind of FEGS assessment to help identify who may be missing from the portfolio of intended users and disentangle what ecological end products might be expanded to attract those user groups. There were certain inherent biases in the shore-based, indirect assessment of use so boaters and anglers were likely underrepresented. However, studies and estuary management programs focusing exclusively on these direct users often miss the more passive experiencers and viewers of the estuary that value these areas for different reasons. Thus, this study sheds light on uses that may be more difficult to quantify with traditional valuation methods. Seasonality and timing were likely additional confounding factors of this study that could be remedied in future FEGS assessments. Conducting monthly surveys, targeting optimal weather conditions, accounting for tide cycles, and prioritizing weekends and other periods of peak recreational use might yield more expansive results for comparison. For example, the mean tidal range in Tillamook Bay is 1.8 m and sampling sites with direct beach access at high vs low tides could make a difference in recreational users observed. Managers may use the results of this study to decide where they would like to invest in more granularity to resolve some of these questions.

Both the TEP and TBEP have implemented large-scale ecosystem restoration projects that intersected multiple habitats and resulted in ecosystem service provision across numerous beneficiaries (Fulford et al. 2016; Shaw and Dundas 2021). Coastal programs elsewhere that may have staff or resource constraints preventing an extensive study of coastal ecosystem services can still proceed with habitat restoration targeting diverse functions, values and species. Recreational beneficiaries who post photos and those observed during surveys appeared to value viewsheds, water and easy access points; actions that improve habitat conditions will likely have a neutral or net positive effect on people seeking a more passive recreational experience. If a goal is to facilitate greater recreational opportunities, land managers and communities might consider adding safe and equitable access options, ideally with water access.

## 5. Conclusion

Our findings indicate that places with diverse landscape features and composite EEPs, which may be more difficult to quantify, are highly valued by recreational beneficiaries. As such, a conflict need not occur between preserving coastal habitat mosaics (e.g. see Sheaves 2009) for biodiversity, species conservation and ecosystem services provision, especially when done proactively with local stakeholders (Austin et al. 2016; Liquete et al. 2016). For programs poised to evaluate FEGS availability, we modeled an approach that takes advantage of existing data and tools, coupled with targeted survey approaches to fully assess the relationship between landscape features and more passive recreational beneficiaries. The approach used in our study focused on shoreline and land-based users, but could be expanded to provide a comprehensive assessment of where people most seek recreational ecosystem services within a given landscape, and also reveal potential physical barriers preventing safe and equitable access. We hope results contribute to comprehensive land use planning efforts that consider recreational FEGS within the context of coastal ecosystem service tradeoffs, sustainability and examining equity at multiple scales.

## Supplementary Material

SI

## Figures and Tables

**Table 1.** Overview of the National Ecosystem Services Classification System (NESCS) Plus three-component structure linking ecological end products to beneficiaries, adapted from EPA, 2020.

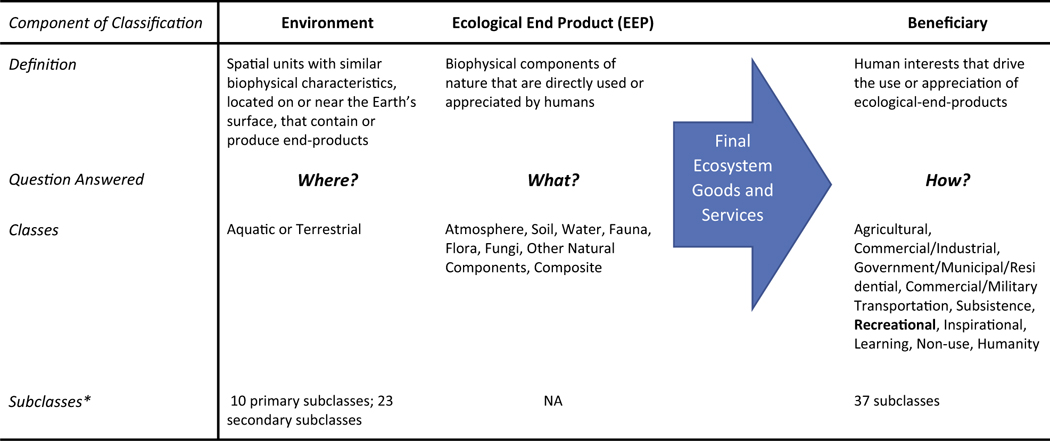

* NESCS accommodates a nested structure whereby the environment and beneficiaries can be characterized with greater specificity. EEP class descriptions and recreational beneficiary subclasses are detailed further in Table 3 and 4, respectively.

**Table 2.** Overview of data sources, their relevance to the study and stated hypotheses, and statistical methods employed to evaluate results.

Data	Description	Scale	Study Relevance	Related Hypotheses*	Statistical Analyses^#^
NESCS Plus 3.0 Webtool	Classification system for describing FEGS supply and demand.	National	Basis for categorizing the FEGS environment, beneficiaries and ecological end products.	*NA*	*NA – informed data characterization*
InVEST Recreation Model output	Model output provided hotspots of recreational activity based on concentration of Flickr photos taken within study-defined area of interest.	Estuary	First step in identifying focal areas for a more in-depth assessment of recreational FEGS uses and users. Recreation ‘hotspots’ informed the selection of SITES in this study.	*NA – Assumption that InVEST Recreation Model results primarily depict areas of recreational FEGS use.*	ArcGIS cluster analysis
Flickr photos	Publicly available images uploaded to the Flickr photo-sharing platform	Site	Photos taken within InVEST grid cells with greatest density of photo-user-days (PUD) were downloaded and content was characterized. The grid cells with the highest PUD count were study SITES.	***H***_***0***_ ***1; H***_***0***_ ***2***	Kruskal-Wallis Rank Sum Test; Dwass-Steel-Crichtlow-Fligner Test for follow-up pairwise comparisons; Flowmap to visualize FEGS beneficiary linkages to ecological end products
Station assessments	Data collected describing the physical attributes and setting of each station	Station	A random selection of five photo coordinates within the approximate 1-km hexagonal grid cell of the SITE were study STATIONS.	***H***_***0***_ ***4; H***_***0***_ ***5; H***_***0***_ ***6***	Moran’s 1 Test for spatial autocorrelation; Nonmetric multi-dimensional scaling (NMDS); Bray-Curtis Dissimilarity Index corrected for binary data; K-means Cluster analysis
User observations	Passive observations of people recreating at each station	Station	The same STATIONS used for assessing onsite characteristics were passively surveyed to evaluate the number or FEGS users and types of recreational activities.	***H***_***0***_ ***3; H***_***0***_ ***7***	Zero-inflated negative binomial (ZINB) mixed-effects models; Akaike Information Criterion (AIC); Tukey Test for follow-up pairwise comparisons of variable levels

* ***H***_***0***_***1***: Flickr images will show a greater diversity of recreational users in Tampa Bay than Tillamook Bay; ***H***_***0***_***2***: Dominant recreational beneficiaries will differ between Tampa and Tillamook bays; ***H***_***0***_***3***: Tampa Bay will have more recreational users observed than Tillamook Bay, but the diversity of recreational beneficiaries will be comparable; ***H***_***0***_***4***: Location (sites and stations) will account for significant variability in the number and suite of recreational beneficiaries; ***H***_***0***_***5***: Stations with more attributes will correspond with greater numbers of recreational uses and beneficiaries; ***H***_***0***_***6***: Stations with similar attributes will have a similar portfolio of recreational beneficiaries; ***H***_***0***_***7***: Onsite user observations will capture a greater diversity of recreational beneficiary subclasses than Flickr data.

# Citations and further details on statistical methods are included in the body of the text under Methods.

**Table 3.** Natural and human-made attributes depicted in Flickr photos or encountered during onsite assessments of selected stations, with the corresponding ecological-end-product (EEP) or access.

Feature	Description^1^	EEP^2^
Fishable or swimmable water	Open water safe for human contact	***water*** *– liquid and solid forms of water including surface water and ground water including components suspended or dissolved in water, which are indicators of water quality*
Navigable water	Open water suitable for operation of a motorized or manually operated boat	
Native plants	Presence of native vegetation	***flora*** *– all plant and unicellular life (for example trees, shrubs, herbs, grasses, ferns, mosses, viruses, bacteria)*
Birds	Presence of birds	***fauna*** *– all animal life (for example, mammals, fish, shellfish, birds,*
Other fauna	Presence of other wildlife or likely habitat	*reptiles, amphibians, insects)*
Created greenspace (*Tampa only*)	Parks, cemeteries, golf courses and the like	***composite*** *– a composite set of specific elements and components of single or multiple environmental classes, including ‘scapes’ (e.g. viewscape, soundscape, scentscape), natural phenomena (e.g. fire, hot springs, geysers), and integrated ecosystems*
Sounds and scents	Natural sounds and smells that may indicate waves/water, wildlife, or native plants	
Viewscape	Natural or semi-natural landscape view	
Beaches and/or dunes	Beaches, dunes, or direct access to beaches or dunes	
Celestial bodies	Typically, the sun or moon, prominently featured in user photographs and considered part of the composite viewscape	
Tidal flats or tide pools	Tidal flats/pools, or direct access to them	
Dock or pier	Presence of a boat launch, dock, or pier providing access to composite features	***site*** *‘****access****’ – these features, when present, readily facilitated human access*
Roadway	A paved or semi-paved road adjacent to a viewscape that allows access by car, bicycle, or another vehicle type	
Railroad (*Tillamook only)*	A railroad adjacent to a viewscape providing human access via train or unofficially serving as a trail	

1 Adapted from (Angradi et al. 2018).

2 EEPs as generally defined by NESCS. Soil and atmosphere were present but excluded because they did not appear to have a direct bearing on site utilization by recreational beneficiaries. ‘Access’ is not an EEP but was added as an important human-made feature class because it can be the nexus facilitating human use (Byczek et al. 2018).

**Table 4.** Description of potentially encountered NESCS recreational beneficiary subclasses.

NESCS Recreational Beneficiary Subclasses	Details*
Anglers	Humans fishing recreationally, including catch-and-release or catch-and-consume activities.
Boaters	Humans using motorized or non-motorized boats to recreate; users fishing from such vessels were categorized as anglers.
Waders, Swimmers, and Divers	Humans recreating in or under the water by wading, swimming, or diving.
Experiencers and Viewers	Broad category of people who enjoy viewing and experiencing biophysical attributes of the environment. People classified as such were observed on shorelines (e.g. intertidal areas, beaches) walking, running, sunbathing, engaging with pets, observing fauna, or generally looking at natural views, as indicated by behavior such as pointing, facing while paused, or taking a picture.

* Descriptions adapted from (Angradi et al. 2018) and FEGS-CS/NESCS. Two NESCS beneficiary classes (i.e. Food Pickers and Gatherers; and Hunters) were not observed during onsite assessments and were excluded from this table and subsequent results.

**Table 5.** Total photo-user-days (PUDs) for each NESCS beneficiary subclass reflected in Flickr images, along with the associated ecological end products (EEPs) depicted in images. For each estuary, the total PUDs are provided, along with the proportion of PUDs for one beneficiary subclass relative to the other beneficiary subclasses.

Beneficiary Subclass	EEPs	PUD Total	
		Tillamook	Tampa
Commercial / Industrial (COM / IND)	atmosphere, composite, fauna, flora, water	15 (0.03)	33 (0.03)
Commercial/Military Transportation (COM / MIL)	atmosphere, water	3 (0.01)	5 (0.01)
Government, Municipal and Residential (GMR)	composite	1 (<0.01)	3 (<0.01)
Recreational (REC)	atmosphere, composite, fauna, flora, water	197 (0.45)	342 (0.36)
non-FEGS		224 (0.51)	561 (0.59)

**Table 6.** Natural and human-made attributes most aligned with each station group, as identified through k-means clustering and nonmetric multidimensional scaling (NMDS) plots (see Figure 6). Most station groups included at least one natural EEP attribute paired and a human-made access point.

	Station Group Alpha	Station Group Beta	Station Group Gamma	Station Group Delta	Station Group Epsilon
**EEP Attributes Viewscape**		✓			✓
Fishable/swimmable water					✓
Navigable water					✓
Natural habitats	✓				
Created greenspace			✓		
Native plants	✓				
**Access**					
Tidal flats w/access*				✓	
Trail	✓				
Roadway			✓		
Railroad					✓

* The single station in Group 4 (i.e. Tillamook Site A, Station #1) included both tidal flats and direct access.

**Table 7.** Comparison of zero-inflated negative binomial (ZINB) models for predicting the observed number of recreational users.

Model Rank	Intercept Terms			Fixed Effects*				Model Summary Statistics			
	Count Model	ZI Model	Dispersion Parameter	SITE	SG	TOD	WTH	df	LogLik	AICc	ΔAIC
1	1.377	−3.164	✓	✓	✓		✓	15	−285.393	607.3	0.00
2	3.025	−2.038	✓	✓			✓	11	−291.798	609.0	1.71
3	1.377	−3.149	✓	✓	✓	✓	✓	16	−285.393	610.2	2.96
4	1.919	−19.770	✓	✓	✓			13	−289.996	610.8	3.51
5	3.446	−2.001	✓	✓				9	−295.433	611.1	3.84
6	3.014	−2.036	✓	✓		✓	✓	12	−291.780	611.6	4.34
7	3.409	−1.961	✓	✓		✓		10	−295.241	613.3	5.99
8	1.923	−19.890	✓	✓	✓	✓		14	−289.970	613.5	6.27
9	2.766	−2.095	✓			✓		5	−303.574	617.9	10.59
10	2.764	−2.091	✓				✓	6	−302.492	618.0	10.72
11	2.827	−2.118	✓			✓	✓	7	−302.178	619.7	12.45
12	2.271	−2.109	✓		✓			8	−301.484	620.7	13.47
13	2.364	−2.268	✓		✓	✓		9	−300.905	622.1	14.79
14	2.247	−2.074	✓		✓		✓	10	−300.702	624.2	16.92
15	2.280	−2.160	✓		✓	✓	✓	11	−300.241	625.9	18.59
16	2.668	−2.026	✓					4			

An ‘✓’ indicates that the term was included in the model corresponding to the given rank.

* Fixed effects in the conditional model: SITE, station group (SG), time of day (TOD) and weather (WTH).

## Data Availability

The data that support the findings of this manuscript will be openly available in ScienceHub at https://catalog.data.gov/dataset/epa-sciencehub.
