# Supplementary material for: Recreational beneficiaries and their landscape dependencies across national estuary program sites: Tillamook Bay (OR) and Tampa Bay (FL), USA
Source: Ecosyst People (Abingdon). Author manuscript; Available in PMC 2024 Nov 15. (PMC10750853; doi:10.1080/26395916.2023.2276756)
Supplement: SI [file NIHMS1946231-supplement-SI.pdf]

Supplementary Materials

Appendix A.

Table A1. Overview of Tillamook Bay and Tampa Bay.

		TBEP TAMPA BAY ESTUARY PROGRAM PARTNERSHIP FOR A HEALTHY BAY
Ecoregion[1]	MEOW: Oregon, Washington, Vancouver Coast and Shelf<br>MENA Level 1: Columbian Pacific<br>MENA Level II: Columbian Shelf<br>MENA Level III: Columbian Neritic	MEOW Northern Gulf of Mexico<br><br>MENA Level I: Northern Gulf of Mexico<br>MENA Level II: Northern Gulf of Mexico Shelf<br>MENA Level III: Western Florida Estuarine Area
Anthropogenic biome[2] of surrounding area	Populated forest	Urban
Approximate estuarine area	34 km^2	1,030 km^2
Average water depth	1.8 m	3.7 m
Surrounding population size[3]	~27,000 people	~2.9 million people
Active seaport(s) and average contribution to local economy	Port of Garibaldi generates ~ US$15 million annually	Ports of Tampa Bay, St. Petersburg, and Port Manatee generate a combined ~ US$15 billion annually
Major industries	agriculture, lumber, tourism, commercial and recreational fishing, seafood processing	defense, avionics, business and information services, manufacturing, tourism, commercial and recreational fishing
Average annual rainfall	224 cm	118 cm
Average temperature	6–16 ^{o}C	18–28 ^{o}C
Estuarine habitats[4]	Salt marshes; aquatic beds; freshwater emergent wetlands; forested wetlands; mudflats	Seagrass meadows; emergent tidal wetlands; mangroves, salt marshes, salt barrens; tidal flats; oyster reef/bars; hard bottom; tidal tributaries, creeks, and rivers; freshwater wetlands; coastal uplands

[1]Based on the Marine Ecoregions of the World (MEOW) classification system (Spalding et al. 2007) and the Marine Ecoregions of North America, MENA (Wilkinson et al. 2009); [2]Ellis and Ramankutty 2008; [3]Population estimates are based on U.S. Census data (census.gov) as of July 1, 2019. Tillamook Bay is wholly within Tillamook County, Oregon and the population estimate is based solely on individuals residing in that county; whereas Tampa Bay is bordered by Hillsborough, Manatee, and Pinellas counties in Florida and the population estimate is the approximate number of people residing in all three counties. [4]Priority estuarine habitats identified in their respective NEP Comprehensive Conservation and Management Plans (TEP 2019, TBEP 2017).

Tillamook Bay

The Tillamook Estuaries Partnership (TEP) was established in 1994 with a focus on building community collaborations to effectively manage estuarine resources throughout the county and find science-based solutions to tackle emerging issues. One of the first actions undertaken by Tillamook County was drafting the Tillamook Bay Comprehensive Conservation and Management Plan (CCMP, Tillamook Bay National Estuary Project 1999) which identified the most pressing issues in the bay, namely: high bacteria concentrations affecting water quality; erosion/sedimentation; declining fish habitat; and flooding. The TEP was later established to not only address these issues, but also to aid in conserving estuarine and watershed habitats throughout Tillamook County. The organization has since brought together industry representatives, community members, and resource agencies to address CCMP goals (TEP 2020). Key actions have included establishing a voluntary water quality monitoring program (active since 1997), removing barriers to fish migration, enhancing riparian vegetation along rivers and streams, restoring tidal wetlands, in-stream habitat enhancement, and completing a climate change vulnerability assessment. In the last two decades, TEP and local partners have completed over 800 habitat-related projects and restored over 550 acres of estuarine habitat (TEP 2019). In 2017, the TEP and its partners completed the Southern Flow Corridor project restoring 443 acres of tidal wetland habitats with concomitant benefits to water quality, salmon habitat, flood mitigation, carbon storage, and the local housing market (Shaw and Dundas 2021). These accomplishments and ongoing efforts to continually improve habitat and water quality, while supporting economic opportunities (TEP 2019), made Tillamook Bay an ideal location for evaluating estuarine natural and man-made attributes facilitating recreational FEGS.

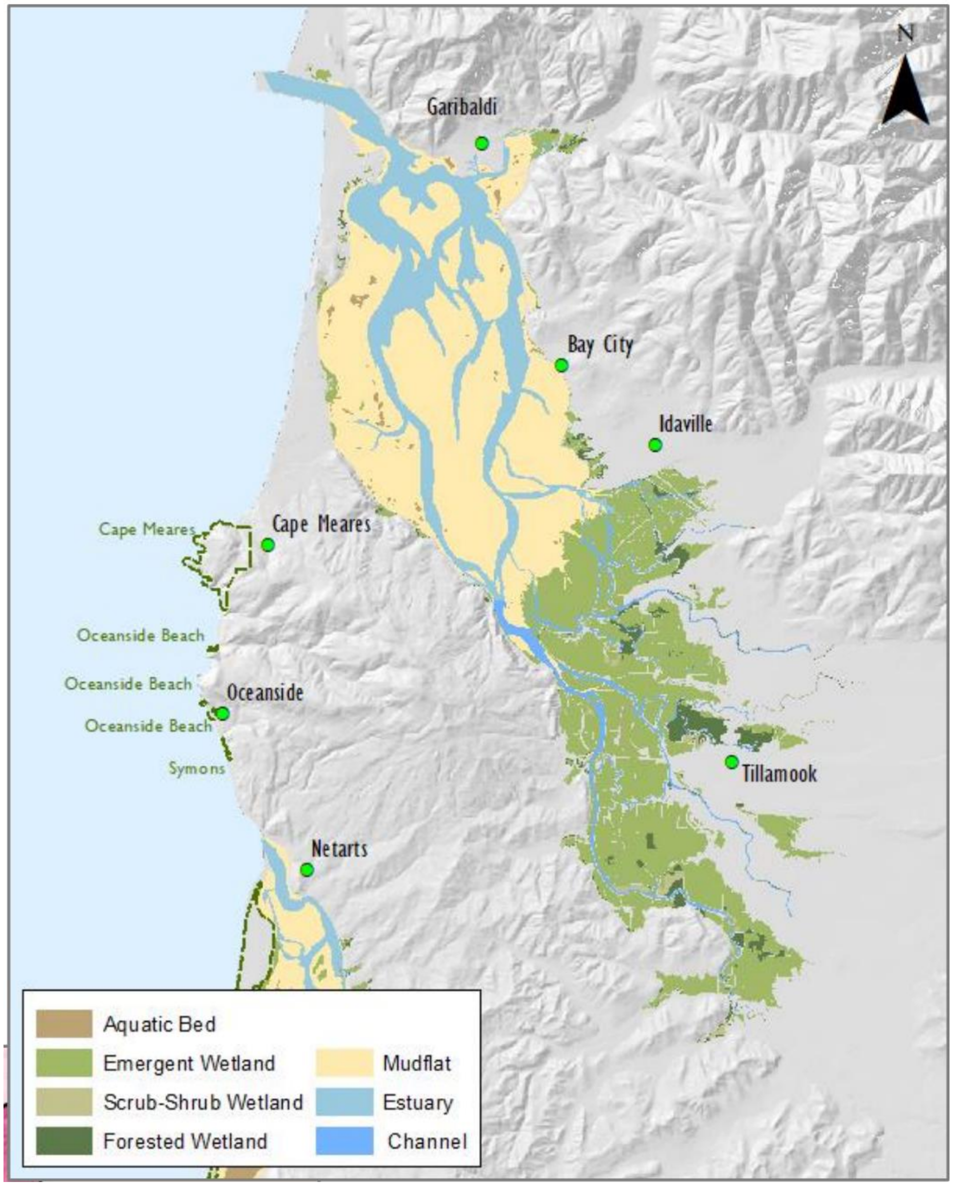

Figure A1. Tillamook Bay habitats based on the Coastal Marine and Estuarine Classification System (CMECS).

<u>Tampa Bay</u>

The Tampa Bay Estuary Program (TBEP) was established in the early 1990s with an initial task to work with local stakeholders and develop a CCMP outlining the major issues and goals. The CCMP was completed by 1996 with a focus on improving water quality and restoring key habitats within the bay (TBEP 1996). Since that time, TBEP has worked extensively with state and federal resource agencies, along with local jurisdictions, to reduce nutrient loads and restore seagrass throughout the bay (TBEP 2017). These combined efforts have resulted in significant reductions in nutrient loads that have led to improved water quality and simultaneous increases in seagrass coverage far exceeding initial restoration goals (Greening and Janicki 2006, Greening et al. 2014, Sherwood et al. 2017). These improvements have a direct bearing on ecosystem services and realized benefits to local residents. For example, restored seagrass,

marsh, and mangrove habitats are estimated to sequester over 36 million kg of carbon per year and remove over 1.2 million kg of nitrogen annually. These nutrient reductions translate to an estimated US$23 million in avoided costs in wastewater treatment and social costs from greenhouse gas emissions (Russell and Greening 2015). Improved habitat has also been linked to fishery production functions, with demonstrated positive effects on recreational fishing opportunities (Yoskowitz and Russell 2015, Fulford et al. 2016a, 2016b). Similar to reported linkages between habitat and fisheries elsewhere (Jordan et al. 2012, Jackson et al. 2015), there was a direct spatial relationship between seagrass areas and the location of recreational anglers in Tampa Bay (Fulford et al. 2016a).

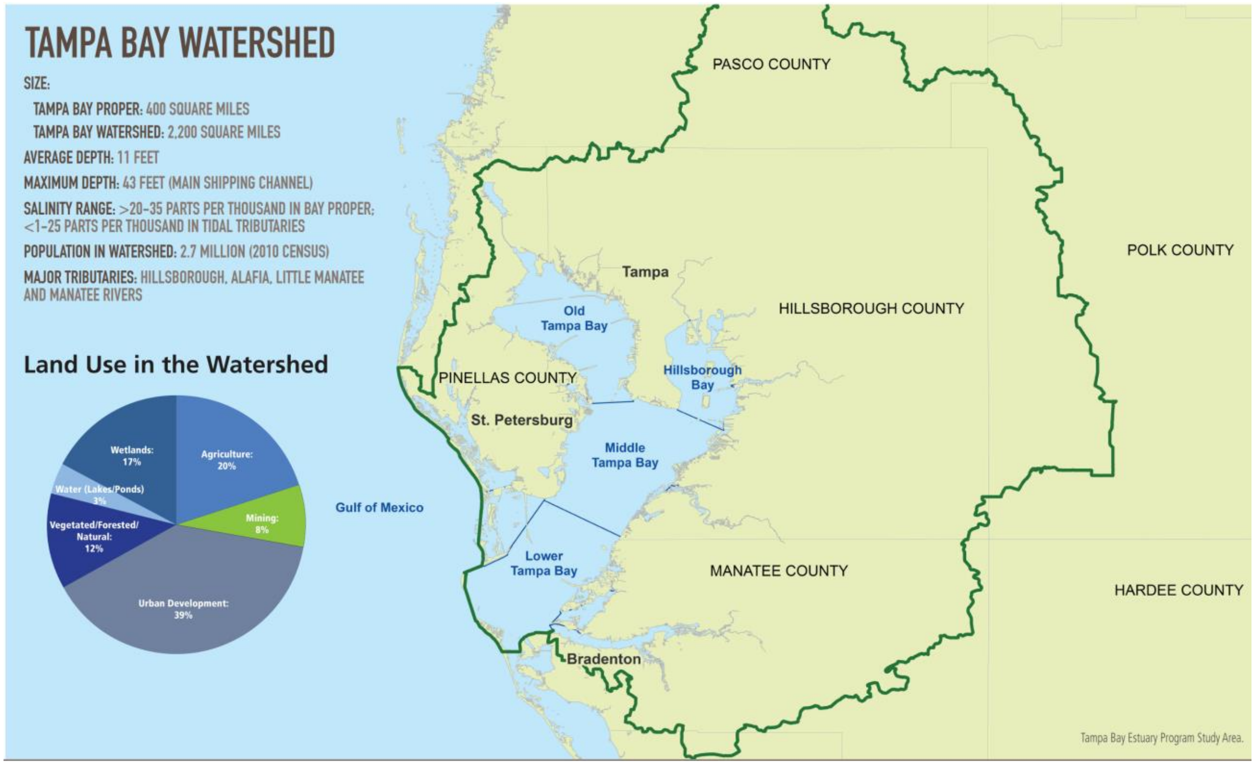

Figure A2. Overview of the Tampa Bay watershed area and prominent land use categories (https://tbep.org/estuary/bay-snapshot/).

References

Ellis EC, Ramankutty N (2008) Putting people in the map: anthropogenic biomes of the world. Front Ecol Environ 6(8):439-447. https://doi.org/10.1890/070062

Fulford RS, Russell M, Rogers JE (2016a) Habitat Restoration from an Ecosystem Goods and Services Perspective: Application of a Spatially Explicit Individual-Based Model. Estuaries and Coasts 39:1801–1815

Fulford R, Yoskowitz D, Russell M, Dantin D, Rogers J (2016b) Habitat and recreational fishing opportunity in Tampa Bay: Linking ecological and ecosystem services to human beneficiaries. Ecosyst Serv 17:64–74

Greening H, Janicki A (2006) Toward reversal of eutrophic conditions in a subtropical estuary: Water quality and seagrass response to nitrogen loading reductions in Tampa Bay, Florida, USA. Environ Manage 38:163–178

Greening H, Janicki A, Sherwood ET, Pribble R, Johansson JOR (2014) Ecosystem responses to long-term nutrient management in an urban estuary: Tampa Bay, Florida, USA. Estuar Coast Shelf Sci 151:A1–A16

Russell M, Greening H (2015) Estimating Benefits in a Recovering Estuary: Tampa Bay, Florida. Estuaries Coasts 38:9–18

Sherwood ET, Greening HS, Johansson JOR, Kaufman K, Raulerson GE (2017) Tampa Bay (Florida, USA): Documenting seagrass recovery since the 1980's and reviewing the benefits. Southeast Geogr 57(3):294–319 https://www.jstor.org/stable/26367669

Spalding MD, Fox HE, Allen GR, Davidson N, Ferdaña ZA, Finlayson M, Halpern BS, Jorge MA, Lombana A, Lourie SA, Martin KD, McManus E, Molnar J, Recchia CA, Robertson J (2007) Marine Ecoregions of the World: A Bioregionalization of Coastal and Shelf Areas. Bioscience 57:573–583

TBEP, Tampa Bay National Estuary Program (1996) Charting the course: The comprehensive conservation and management plan for Tampa Bay. St. Petersburg, FL

TBEP (2017) Charting the course: The comprehensive conservation and management plan for Tampa Bay. August 2017 Revision. St. Petersburg, FL https://indd.adobe.com/view/cf7b3c48-d2b2-4713-921c-c2a0d4466632. Last accessed January 30, 2022

TEP, Tillamook Estuaries Partnership (2019) Tillamook Estuaries Partnership's Comprehensive Conservation and Management Plan. CCMP Update. Tillamook Bay National Estuary Program, Garibaldi, OR. Completed under U.S. Environmental Protection Agency Cooperative Agreement 01J04301.

TEP, Tillamook Estuaries Partnership (2020) 2020 State of the Bays. Tillamook Bay National Estuary Program, Garibaldi, OR. https://www.tbnep.org/reports-publications/stateofbays-web-final-1609.pdf

Tillamook Bay National Estuary Project (1999) Tillamook Bay Comprehensive Conservation and Management Plan. Tillamook Bay National Estuary Project, Garibaldi, OR. Completed under U.S. Environmental Protection Agency Cooperative Agreement CE-980127-01

Wilkinson T, Wiken E, Creel J, Hourigan T, Agardy T, Herrmann H, Janishevski L, Madden C, Morgan L, Padilla M (2009) Marine Ecoregions of North America.

Appendix B.

Time Trials

Time trials were conducted in Tillamook Bay to inform a reasonable period of time for documenting station utilization by potential beneficiaries. A randomly selected station was selected per site and two observers counted the number of people visiting each station over a 2-hour time period.

Table B1. Raw data from Tillamook Bay time trials.

Date	Site	Station	Observer	Period	Time of Observation	Person Count	Notes
8/7/2018	A	A0	CJL	1510_1710	15:16	6	
8/7/2018	A	A0	CJL	1510_1710	15:18	2	
8/7/2018	A	A0	CJL	1510_1710	15:25	4	
8/7/2018	A	A0	CJL	1510_1710	15:27	29	scenic train
8/7/2018	A	A0	CJL	1510_1710	15:29	2	
8/7/2018	A	A0	CJL	1510_1710	15:35	2	
8/7/2018	A	A0	CJL	1510_1710	15:39	5	
8/7/2018	A	A0	CJL	1510_1710	15:40	2	
8/7/2018	A	A0	CJL	1510_1710	15:41	2	
8/7/2018	A	A0	CJL	1510_1710	15:44	6	
8/7/2018	A	A0	CJL	1510_1710	15:46	2	
8/7/2018	A	A0	CJL	1510_1710	15:47	4	
8/7/2018	A	A0	CJL	1510_1710	15:53	2	
8/7/2018	A	A0	CJL	1510_1710	15:56	4	
8/7/2018	A	A0	CJL	1510_1710	16:00	2	
8/7/2018	A	A0	CJL	1510_1710	16:02	2	
8/7/2018	A	A0	CJL	1510_1710	16:04	11	
8/7/2018	A	A0	CJL	1510_1710	16:06	2	
8/7/2018	A	A0	CJL	1510_1710	16:07	4	
8/7/2018	A	A0	CJL	1510_1710	16:10	2	
8/7/2018	A	A0	CJL	1510_1710	16:11	2	
8/7/2018	A	A0	CJL	1510_1710	16:12	2	
8/7/2018	A	A0	CJL	1510_1710	16:13	2	
8/7/2018	A	A0	CJL	1510_1710	16:15	2	
8/7/2018	A	A0	CJL	1510_1710	16:16	1	
8/7/2018	A	A0	CJL	1510_1710	16:18	4	
8/7/2018	A	A0	CJL	1510_1710	16:20	2	
8/7/2018	A	A0	CJL	1510_1710	16:25	2	
8/7/2018	A	A0	CJL	1510_1710	16:26	4	
8/7/2018	A	A0	CJL	1510_1710	16:28	2	
8/7/2018	A	A0	CJL	1510_1710	16:30	4	

Date	Site	Station	Observer	Period	Time of Observation	Person Count	Notes
8/7/2018	A	A0	CJL	1510_1710	16:32	1	
8/7/2018	A	A0	CJL	1510_1710	16:36	2	
8/7/2018	A	A0	CJL	1510_1710	16:37	2	
8/7/2018	A	A0	CJL	1510_1710	16:38	3	
8/7/2018	A	A0	CJL	1510_1710	16:41	2	
8/7/2018	A	A0	CJL	1510_1710	16:43	2	
8/7/2018	A	A0	CJL	1510_1710	16:45	3	
8/7/2018	A	A0	CJL	1510_1710	16:49	2	
8/7/2018	A	A0	CJL	1510_1710	16:50	2	
8/7/2018	A	A0	CJL	1510_1710	16:52	2	
8/7/2018	A	A0	CJL	1510_1710	16:58	4	
8/7/2018	A	A0	CJL	1510_1710	17:06	2	
8/7/2018	B	B4	CJL	1244_1444	12:47	2	
8/7/2018	B	B4	CJL	1244_1444	12:50	2	
8/7/2018	B	B4	CJL	1244_1444	12:53	2	
8/7/2018	B	B4	CJL	1244_1444	12:56	6	
8/7/2018	B	B4	CJL	1244_1444	13:01	2	
8/7/2018	B	B4	CJL	1244_1444	13:05	2	
8/7/2018	B	B4	CJL	1244_1444	13:15	1	
8/7/2018	B	B4	CJL	1244_1444	13:16	2	
8/7/2018	B	B4	CJL	1244_1444	13:17	3	
8/7/2018	B	B4	CJL	1244_1444	13:18	4	
8/7/2018	B	B4	CJL	1244_1444	13:19	1	
8/7/2018	B	B4	CJL	1244_1444	13:20	2	
8/7/2018	B	B4	CJL	1244_1444	13:21	2	
8/7/2018	B	B4	CJL	1244_1444	13:25	1	
8/7/2018	B	B4	CJL	1244_1444	13:26	3	
8/7/2018	B	B4	CJL	1244_1444	13:27	2	
8/7/2018	B	B4	CJL	1244_1444	13:41	2	
8/7/2018	B	B4	CJL	1244_1444	14:01	2	
8/7/2018	B	B4	CJL	1244_1444	14:04	2	
8/7/2018	B	B4	CJL	1244_1444	14:07	2	
8/7/2018	B	B4	CJL	1244_1444	14:13	2	
8/7/2018	B	B5	CJL	1244_1445	14:14	0	
8/7/2018	B	B6	CJL	1244_1446	14:24	0	
8/7/2018	B	B4	CJL	1244_1444	14:25	5	
8/7/2018	B	B4	CJL	1244_1444	14:26	2	
8/7/2018	B	B4	CJL	1244_1444	14:37	2	
8/7/2018	B	B4	CJL	1244_1444	14:42	1	
8/7/2018	C	C4	CJL	1015_1215	10:17	2	
8/7/2018	C	C4	CJL	1015_1215	10:24	1	

Date	Site	Station	Observer	Period	Time of Observation	Person Count	Notes
8/7/2018	C	C4	CJL	1015_1215	10:31	2	
8/7/2018	C	C4	CJL	1015_1215	10:35	0	
8/7/2018	C	C4	CJL	1015_1215	10:45	0	
8/7/2018	C	C4	CJL	1015_1215	10:55	0	
8/7/2018	C	C4	CJL	1015_1215	11:05	2	
8/7/2018	C	C4	CJL	1015_1215	11:18	1	
8/7/2018	C	C4	CJL	1015_1215	11:35	4	
8/7/2018	C	C4	CJL	1015_1215	11:41	2	
8/7/2018	C	C4	CJL	1015_1215	11:43	2	
8/7/2018	C	C4	CJL	1015_1215	11:47	2	
8/7/2018	C	C4	CJL	1015_1215	11:56	2	
8/7/2018	A	A0	NL	1510_1710	15:15	2	
8/7/2018	A	A0	NL	1510_1710	15:17	4	
8/7/2018	A	A0	NL	1510_1710	15:19	2	
8/7/2018	A	A0	NL	1510_1710	15:25	4	
8/7/2018	A	A0	NL	1510_1710	15:27	29	scenic train
8/7/2018	A	A0	NL	1510_1710	15:29	2	
8/7/2018	A	A0	NL	1510_1710	15:35	2	
8/7/2018	A	A0	NL	1510_1710	15:39	3	
8/7/2018	A	A0	NL	1510_1710	15:40	6	
8/7/2018	A	A0	NL	1510_1710	15:41	2	
8/7/2018	A	A0	NL	1510_1710	15:44	4	
8/7/2018	A	A0	NL	1510_1710	15:46	4	
8/7/2018	A	A0	NL	1510_1710	15:47	4	
8/7/2018	A	A0	NL	1510_1710	15:53	2	
8/7/2018	A	A0	NL	1510_1710	15:56	8	
8/7/2018	A	A0	NL	1510_1710	16:00	5	
8/7/2018	A	A0	NL	1510_1710	16:02	2	
8/7/2018	A	A0	NL	1510_1710	16:03	11	
8/7/2018	A	A0	NL	1510_1710	16:07	6	
8/7/2018	A	A0	NL	1510_1710	16:10	2	
8/7/2018	A	A0	NL	1510_1710	16:12	2	
8/7/2018	A	A0	NL	1510_1710	16:14	2	
8/7/2018	A	A0	NL	1510_1710	16:15	2	
8/7/2018	A	A0	NL	1510_1710	16:16	1	
8/7/2018	A	A0	NL	1510_1710	16:18	4	
8/7/2018	A	A0	NL	1510_1710	16:26	4	
8/7/2018	A	A0	NL	1510_1710	16:28	4	
8/7/2018	A	A0	NL	1510_1710	16:30	3	
8/7/2018	A	A0	NL	1510_1710	16:31	2	
8/7/2018	A	A0	NL	1510_1710	16:32	2	

Date	Site	Station	Observer	Period	Time of Observation	Person Count	Notes
8/7/2018	A	A0	NL	1510_1710	16:37	2	
8/7/2018	A	A0	NL	1510_1710	16:41	4	
8/7/2018	A	A0	NL	1510_1710	16:43	2	
8/7/2018	A	A0	NL	1510_1710	16:52	2	
8/7/2018	A	A0	NL	1510_1710	16:57	4	
8/7/2018	A	A0	NL	1510_1710	17:06	2	
8/7/2018	B	B4	NL	1244_1444	12:44	2	
8/7/2018	B	B4	NL	1244_1444	12:50	2	
8/7/2018	B	B4	NL	1244_1444	12:53	2	
8/7/2018	B	B4	NL	1244_1444	12:55	6	
8/7/2018	B	B4	NL	1244_1444	13:00	2	
8/7/2018	B	B4	NL	1244_1444	13:05	2	
8/7/2018	B	B4	NL	1244_1444	13:15	1	
8/7/2018	B	B4	NL	1244_1444	13:16	3	
8/7/2018	B	B4	NL	1244_1444	13:17	3	
8/7/2018	B	B4	NL	1244_1444	13:18	5	
8/7/2018	B	B4	NL	1244_1444	13:19	2	
8/7/2018	B	B4	NL	1244_1444	13:20	2	
8/7/2018	B	B4	NL	1244_1444	13:21	2	
8/7/2018	B	B4	NL	1244_1444	13:26	6	
8/7/2018	B	B4	NL	1244_1444	13:41	1	
8/7/2018	B	B4	NL	1244_1444	14:01	2	
8/7/2018	B	B4	NL	1244_1444	14:04	2	
8/7/2018	B	B4	NL	1244_1444	14:07	2	
8/7/2018	B	B4	NL	1244_1444	14:13	2	
8/7/2018	B	B4	NL	1244_1444	14:14	0	
8/7/2018	B	B4	NL	1244_1444	14:24	0	
8/7/2018	B	B4	NL	1244_1444	14:25	5	
8/7/2018	B	B4	NL	1244_1444	14:26	2	
8/7/2018	B	B4	NL	1244_1444	14:36	2	
8/7/2018	B	B4	NL	1244_1444	14:42	1	
8/7/2018	C	C4	NL	1015_1215	10:24	1	
8/7/2018	C	C4	NL	1015_1215	10:31	2	
8/7/2018	C	C4	NL	1015_1215	10:35	0	
8/7/2018	C	C4	NL	1015_1215	10:45	0	
8/7/2018	C	C4	NL	1015_1215	10:55	0	
8/7/2018	C	C4	NL	1015_1215	11:06	2	
8/7/2018	C	C4	NL	1015_1215	11:15	0	
8/7/2018	C	C4	NL	1015_1215	11:19	3	
8/7/2018	C	C4	NL	1015_1215	11:22	2	
8/7/2018	C	C4	NL	1015_1215	11:35	6	

Date	Site	Station	Observer	Period	Time of Observation	Person Count	Notes
8/7/2018	C	C4	NL	1015_1215	11:43	2	
8/7/2018	C	C4	NL	1015_1215	11:47	2	
8/7/2018	C	C4	NL	1015_1215	11:56	4	

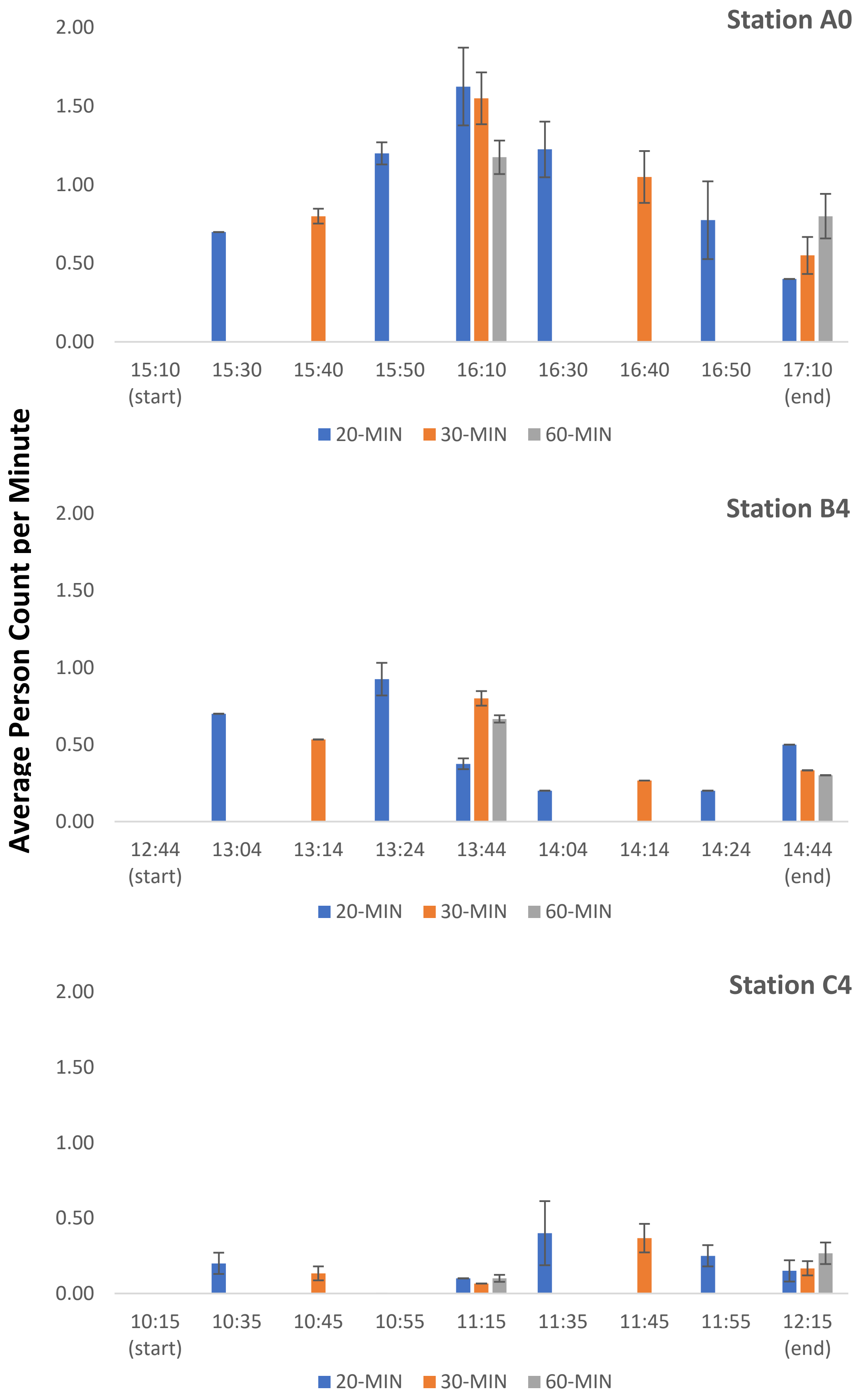
Average Person Count per Minute
Station A0
2.00
1.50
1.00
0.50
0.00
15:10 (start)
15:30
15:40
15:50
16:10
16:30
16:40
16:50
17:10 (end)
20-MIN
30-MIN
60-MIN
Station B4
2.00
1.50
1.00
0.50
0.00
12:44 (start)
13:04
13:14
13:24
13:44
14:04
14:14
14:24
14:44 (end)
20-MIN
30-MIN
60-MIN
Station C4
2.00
1.50
1.00
0.50
0.00
10:15 (start)
10:35
10:45
10:55
11:15
11:35
11:45
11:55
12:15 (end)
20-MIN
30-MIN
60-MIN

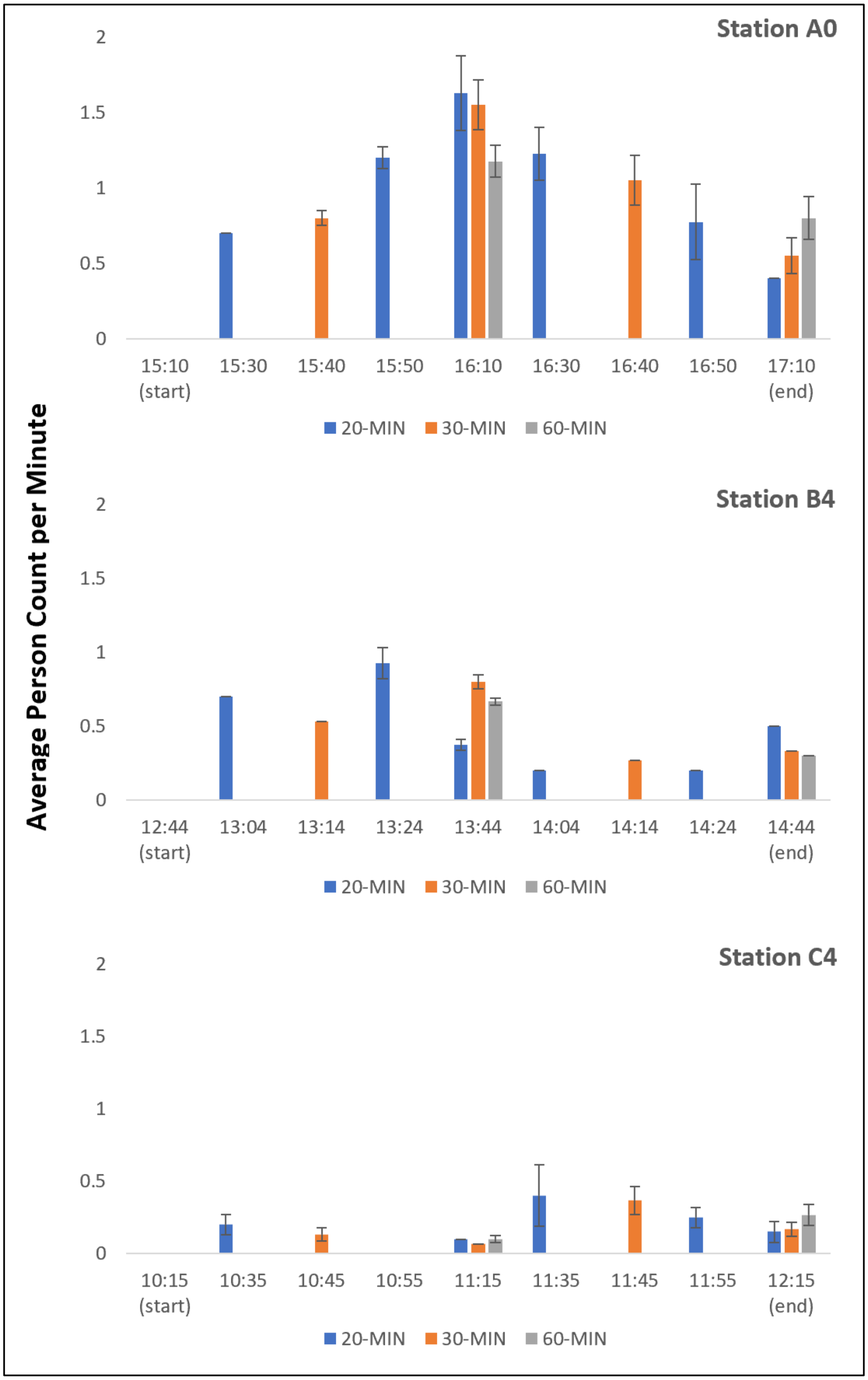

Figure B1. Average person count per minute at three Tillamook stations by 20, 30, and 60-minute time increments during the two-hour time trial.

Appendix C.

Moran’s I Spatial Autocorrelation Test Results

We tested for potential spatial autocorrelation between stations based on the number of observed users. We ran the test for the combined dataset with stations from both estuaries, and then ran the test for each estuary independently. There was no evidence to reject the null hypothesis, whether the stations were grouped by estuary in advance, or not. Results were identical using the “ape” package in R (Paradis et al. 2004).

Reference

Paradis E, Claude J, Strimmer K (2004) APE: analyses of phylogenetics and evolution in R language. J Bioinform 20: 289–290

Spatial Autocorrelation Report

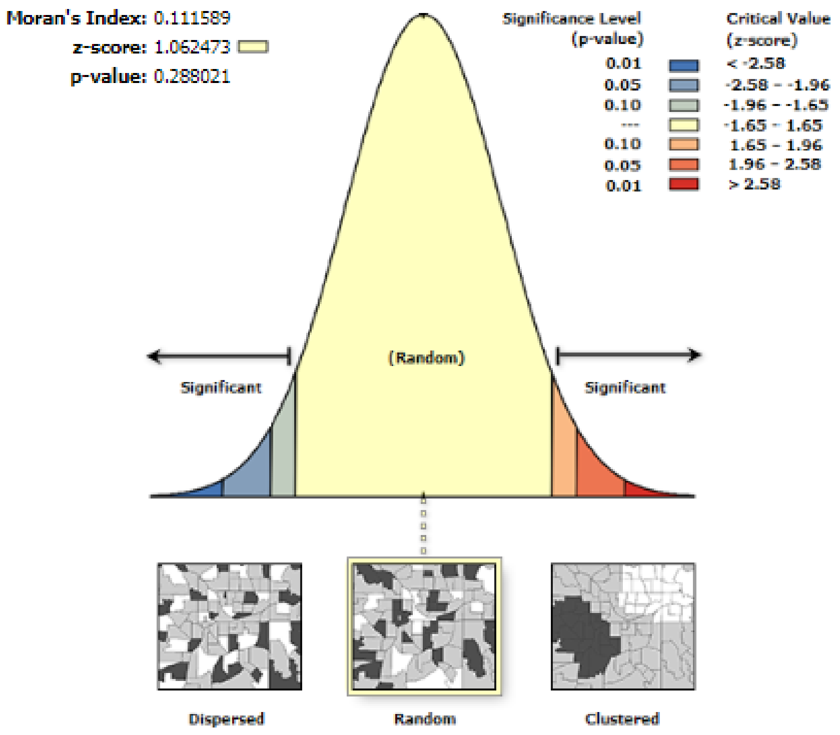

Given the z-score of 1.06247304311, the pattern does not appear to be significantly different than random.

Global Moran's I Summary

**Moran's Index:**	0.111589
**Expected Index:**	-0.034483
**Variance:**	0.018902
**z-score:**	1.062473
**p-value:**	0.288021

Dataset Information

**Input Feature Class:**	TillTampa_stations_projected
**Input Field:**	ALL_USERS_
**Conceptualization:**	INVERSE_DISTANCE
**Distance Method:**	EUCLIDEAN
**Row Standardization:**	True
**Distance Threshold:**	6354080.8468 Meters
**Weights Matrix File:**	None
**Selection Set:**	False

Spatial Autocorrelation Report

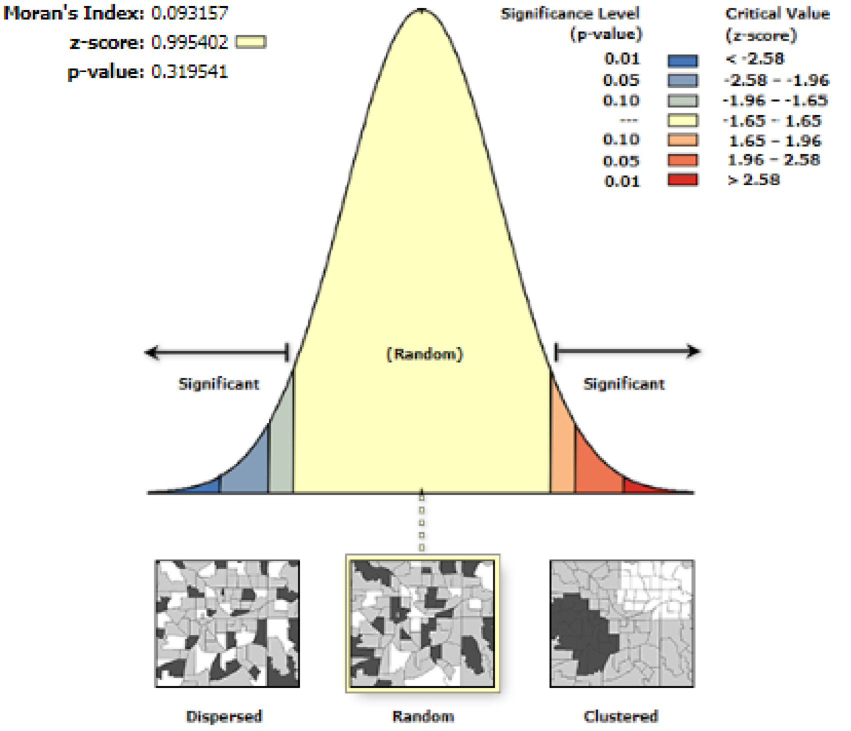

Given the z-score of 0.995402040818, the pattern does not appear to be significantly different than random.

Global Moran's I Summary

**Moran's Index:**	0.093157
**Expected Index:**	-0.071429
**Variance:**	0.027339
**z-score:**	0.995402
**p-value:**	0.319541

Dataset Information

**Input Feature Class:**	Tillamook_stations_projected
**Input Field:**	ALL_USERS_
**Conceptualization:**	INVERSE_DISTANCE
**Distance Method:**	EUCLIDEAN
**Row Standardization:**	True
**Distance Threshold:**	7129.2298 Meters
**Weights Matrix File:**	None
**Selection Set:**	False

Spatial Autocorrelation Report

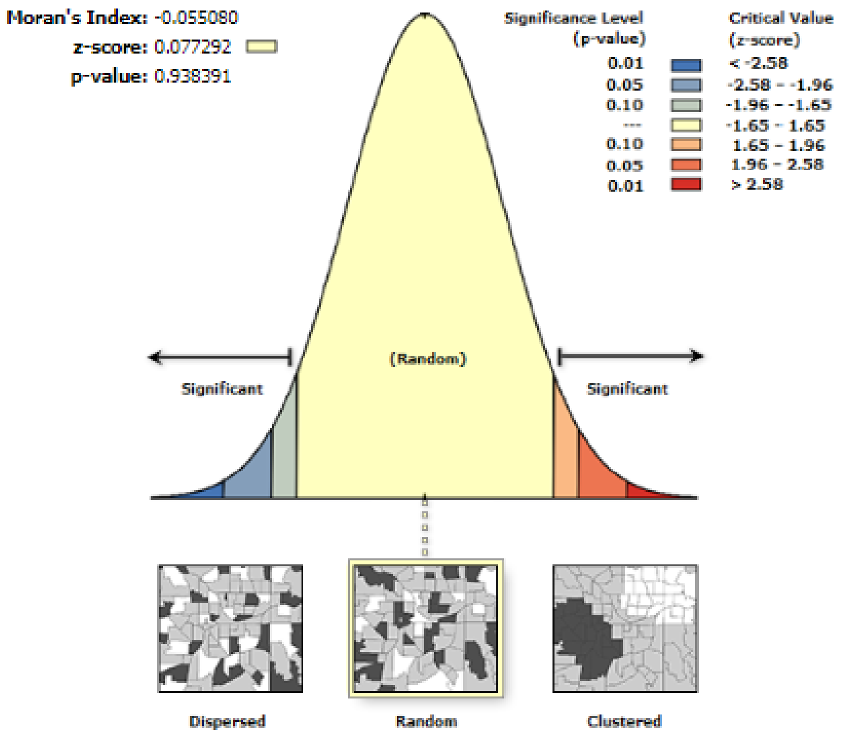

Given the z-score of 0.0772921025976, the pattern does not appear to be significantly different than random.

Global Moran's I Summary

**Moran's Index:**	-0.055080
**Expected Index:**	-0.071429
**Variance:**	0.044740
**z-score:**	0.077292
**p-value:**	0.938391

Dataset Information

**Input Feature Class:**	Tampa_stations_projected
**Input Field:**	ALL_USERS_
**Conceptualization:**	INVERSE_DISTANCE
**Distance Method:**	EUCLIDEAN
**Row Standardization:**	True
**Distance Threshold:**	58767.6513 Meters
**Weights Matrix File:**	None
**Selection Set:**	False
